# Supplementary material for: Human cell based directed evolution of adenine base editors with improved efficiency
Source: Nat Commun. 2021 Oct 8;12:5897. doi: 10.1038/s41467-021-26211-0 (PMC8501064; doi:10.1038/s41467-021-26211-0)
Supplement: Supplementary file 1 — Supplementary Information [file 41467_2021_26211_MOESM1_ESM.pdf]

Supplementary Information

Human cell based directed evolution of adenine base editors with improved efficiency

Junhao Fu[1&], Qing Li[2&], Xiaoyu Liu[1&], Tianxiang Tu[1&], Xiujuan Lv[1], Jineng Lv[1], Xidi Yin[2], Zongming Song[1,3], Jia Qu[1], Jinwei Zhang[4], Jinsong Li[2*], and Feng Gu[1*]

[1]School of Ophthalmology and Optometry, Eye Hospital, Wenzhou Medical University, State Key Laboratory and Key Laboratory of Vision Science, Ministry of Health and Zhejiang Provincial Key Laboratory of Ophthalmology and Optometry, Wenzhou, Zhejiang, China

[2]State Key Laboratory of Cell Biology, Shanghai Key Laboratory of Molecular Andrology, Shanghai Institute of Biochemistry and Cell Biology, Center for Excellence in Molecular Cell Science, Chinese Academy of Sciences, Shanghai, China

[3]Henan Eye Hospital, Henan Eye Institute, Henan Provincial People's Hospital and People's Hospital of Zhengzhou University and People's Hospital of Henan University, Zhengzhou, Henan, China.

[4]Laboratory of Molecular Biology, National Institute of Diabetes and Digestive and Kidney Diseases, National Institutes of Health, Bethesda, Maryland, USA.

[&]These authors contributed equally to this work.

* **Correspondence:**

Feng Gu, **E-mail:** gufengw@gmail.com, Jinsong Li, **E-mail:** jsli@sibcb.ac.cn

Supplementary data	
Supplementary Figure 1	Engineering *EGFP* gene for the optimization of ABE.
Supplementary Figure 2	The fluorescence intensity of wild-type and engineered *EGFP*.
Supplementary Figure 3	Generation and screening of the libraries.
Supplementary Figure 4	The editing efficiencies of ABE variants at A12.
Supplementary Figure 5	Flow cytometric analysis showing boosted editing efficiency of three ABE variants.
Supplementary Figure 6	Schematic of NG-ABEmax variants structures.
Supplementary Figure 7	Comparison of the activity of NG-ABEmax and NG-ABEmax variants at endogenous genomic sites.
Supplementary Figure 8	Variants harboring a strong basic residue at position 127 in TadA boosting the editing efficiency.
Supplementary Figure 9	Editing efficiencies of NG-ABEmax-KR.
Supplementary Figure 10	Increased editing window of NG-ABEmax-KR at endogenous genomic sites.
Supplementary Figure 11	Sequence information of mice generated with NG-ABEmax-KR.
Supplementary Figure 12	Editing efficiencies of NG-ABEmax or NG-ABEmax-KR in mouse embryos.
Supplementary Figure 13	Off-target of NG-ABEmax and NG-ABEmax-KR at endogenous genomic sites.
Supplementary Figure 14	Off-target of NG-ABEmax and NG-ABEmax-KR at mouse *Try* gene.
Supplementary Figure 15	Higher ratio of on-target to off-target editing of NG-ABEmax-KR at human endogenous gene and mouse *Try* gene.
Supplementary Figure 16	RNA off-target editing of NG-ABEmax-KR and variants with two mutations (E59A in TadA, V106W in TadA*).
Supplementary Figure 17	Base editing efficiencies for sgRNA started with different first nucleotide.
Supplementary Figure 18	Activity, protein expression levels and nuclear localization of ABE variants.
Supplementary Table 1	Mutations information of randomly picked variants.
Supplementary Table 2	Summary of mutations information.
Supplementary Table 3	No toxic effect of NG-ABEmax-KR for mouse development.
Supplementary Table 4	Primers used to generate different EGFP variants.
Supplementary Table 5	Primers used to generate sgRNA plasmids targeting different sites of *dEGFP1* or *dEGFP2*.
Supplementary Table 6	Primers used to generate sgRNA plasmid targeting MCS site in the HEK293-PME cell line.
Supplementary Table 7	Primers used to generate sgRNA plasmids targeting different genomic loci.
Supplementary Table 8	Primers used to identify key mutations for L1-60, L1-98 and L2-156.
Supplementary Table 9	Primers used to generate NG-ABEmax-R^{127} and NG-ABEmax H^{127}.
Supplementary Table 10	Primers used to generate 2 NG-ABEmax variants libraries.
Supplementary Table 11	Primers used for fragments amplification.
Supplementary Table 12	Primers used for next-generation sequencing.
Supplementary Table 13	The raw screening data of Library 1.
Supplementary Table 14	The raw screening data of Library 2.

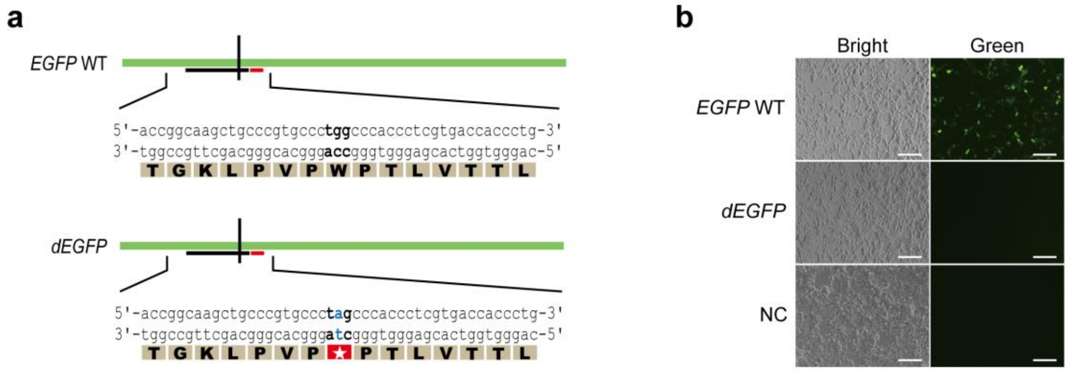

Supplementary Fig. 1 Engineering *EGFP* gene for ABE optimization.

a A single base mutation introducing stop codon (TAG) to inactivate *EGFP*. The stop codon was highlighted with red star. **b** HEK-293 cells were transfected with dead *EGFP* encoding plasmids (200 ng), and images were obtained at 48 hr post-transfection. NC represents the negative control. Scale bar, 10 μm.

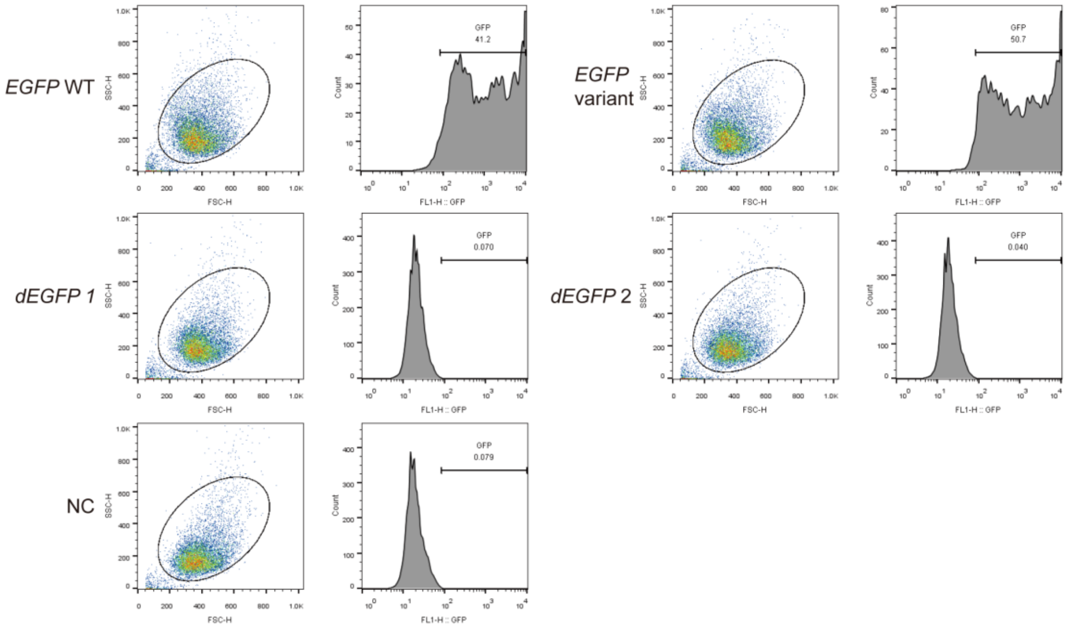

Supplementary Fig. 2 The fluorescence intensity of wild-type and engineered *EGFP*.

The HEK-293 cells were transfected with the plasmids coding for *EGFP* (wild-type) and *EGFP* mutants. The flow cytometry analysis was performed to obtain the percent of *EGFP* positive cells.

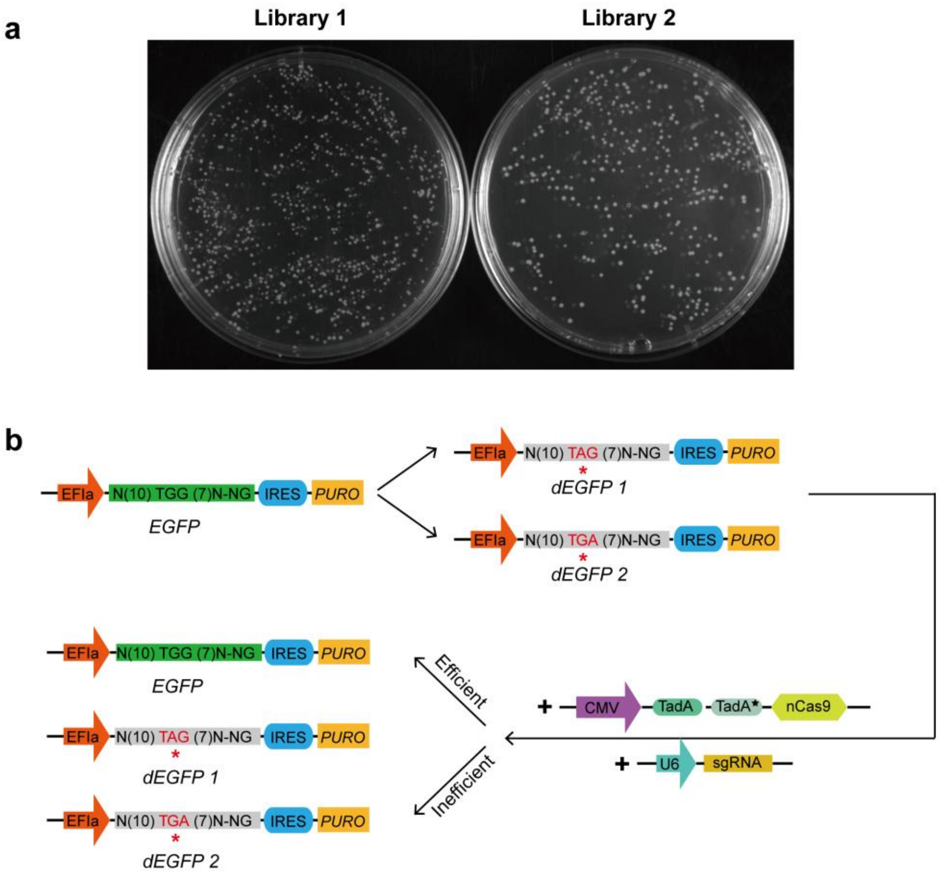

Supplementary Fig. 3 Generation and screening of the libraries.

a There are two libraries (library 1 and library 2) generated in this study. Each library contains two plates; here we only show one plate from each library. **b** The screening strategy of variants.

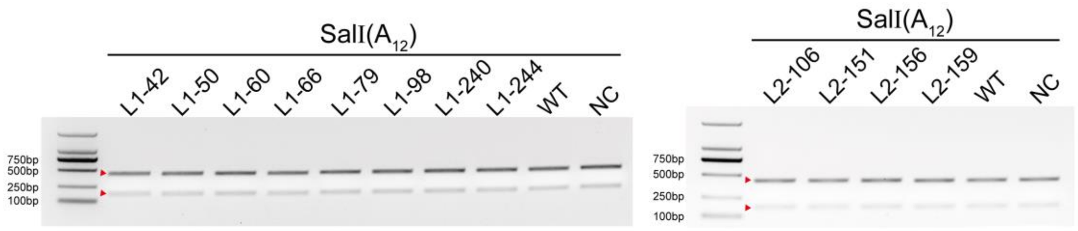

Supplementary Fig. 4 The editing efficiencies of ABE variants at A12.

Agarose gel electrophoresis results of testing editing efficiency of NG-ABEmax variants with SalI. Cleaved bands from SalI are labeled with red triangle. The amplicon is 606-bp. WT and NC represent wild-type NG-ABEmax and no plasmid, respectively.

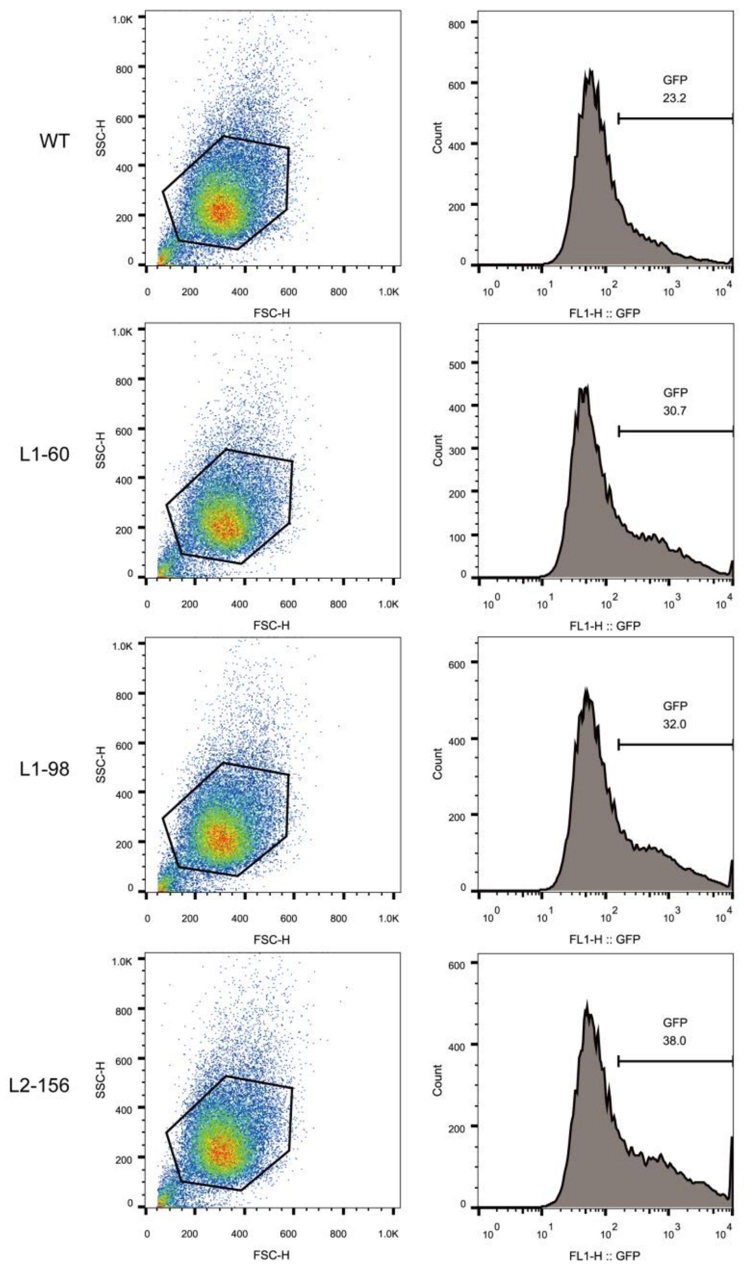

Supplementary Fig. 5 Flow cytometric analysis showing boosted editing efficiency of three ABE variants.

Flow cytometric analysis of three ABE variants at A7. GFP represents the positive signal (percentage).

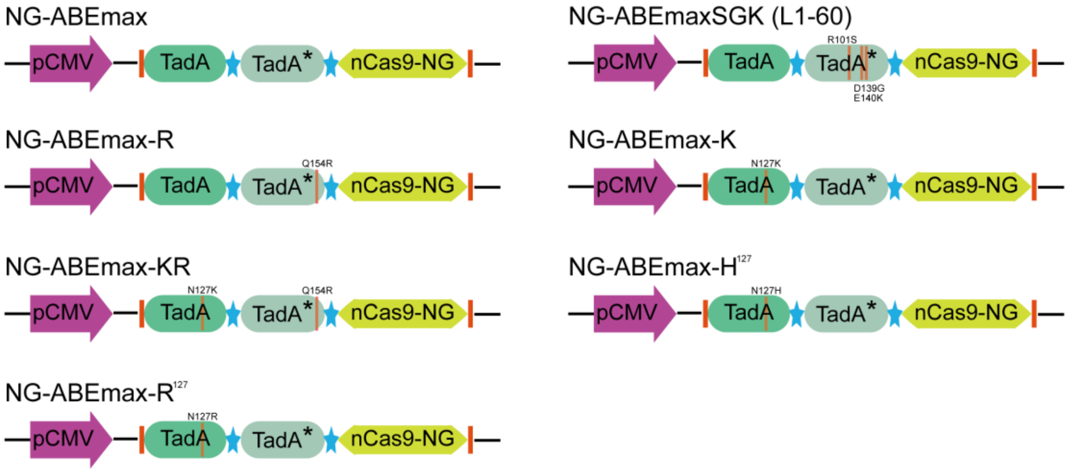

Supplementary Fig. 6 Schematic of NG-ABEmax variants structures.

Two small orange rectangular boxes and pentagrams represent nuclear localization signals and 32-AA linkers, respectively.

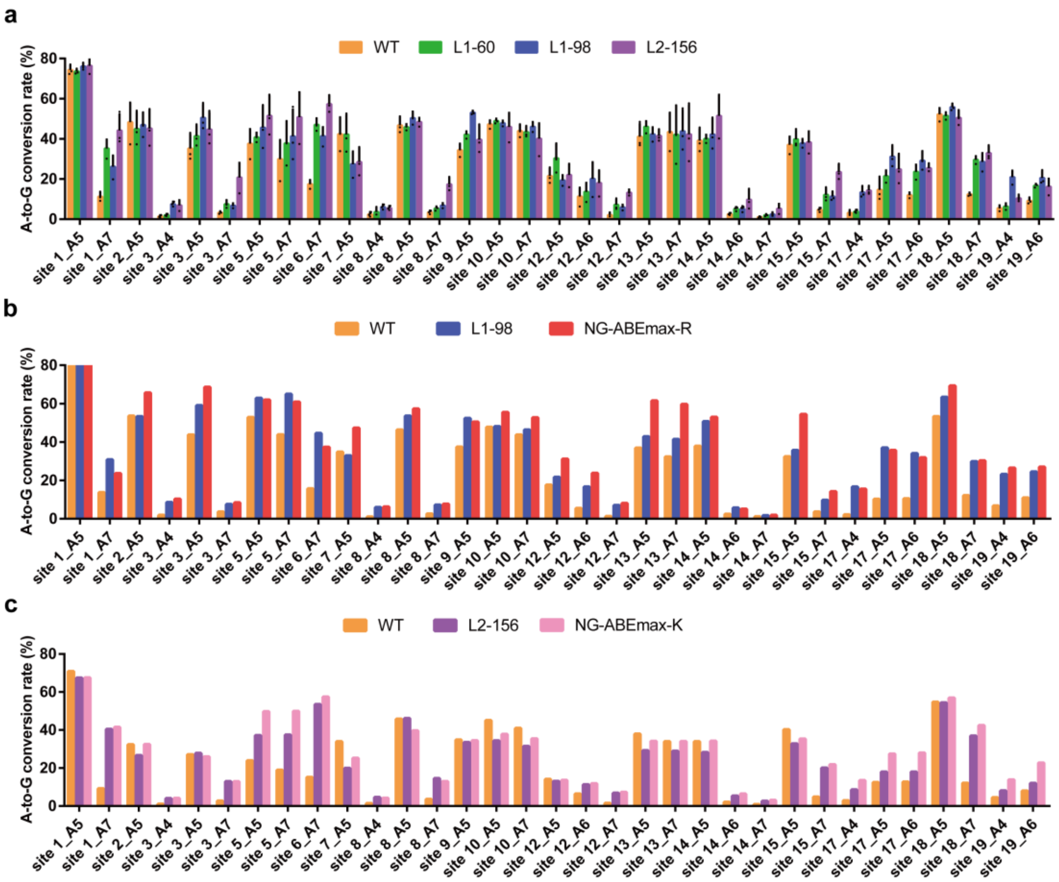

Supplementary Fig. 7 Comparison of the activity of NG-ABEmax and NG-ABEmax variants at endogenous genomic sites.

a Comparison of the activity of NG-ABEmax and NG-ABEmax variants (L1-60, L1-98, L2-156); Error bars indicate mean ± s.d. ($n = 3$ independent experiments). **b** Comparison of the activity of NG-ABEmax, L1-98 and NG-ABEmax-R; **c** Comparison of the activity of NG-ABEmax, L2-156 and NG-ABEmax-K. WT represents wild-type NG-ABEmax.

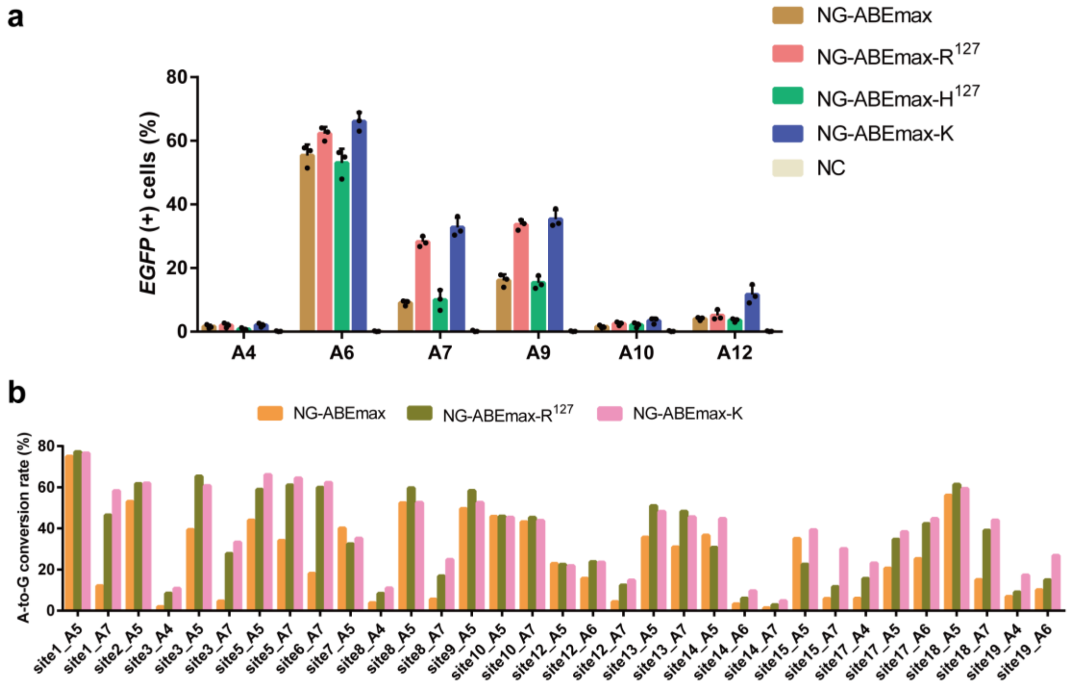

Supplementary Fig. 8 Variants harboring a strong basic residue at position 127 in TadA boosting the editing efficiency.

a The editing efficiencies of three variants harboring different basic residues (H, K and R) at position 127 in TadA via EGFP-based reporter system. EGFP positive cells were quantified by flow cytometry. NC represents the negative control. Error bars indicate mean ± s.d. (*n* = 3 independent experiments). **b** The editing efficiencies of NG-ABEmax, NG-ABEmax-R^{127} and NG-ABEmax-K and at endogenous genomic sites.

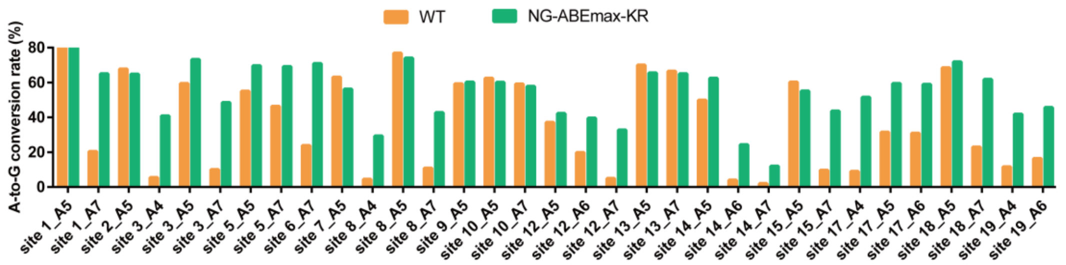

Supplementary Fig. 9 Editing efficiencies of NG-ABEmax-KR.

Boosted editing efficiencies of NG-ABEmax-KR at endogenous genomic sites.

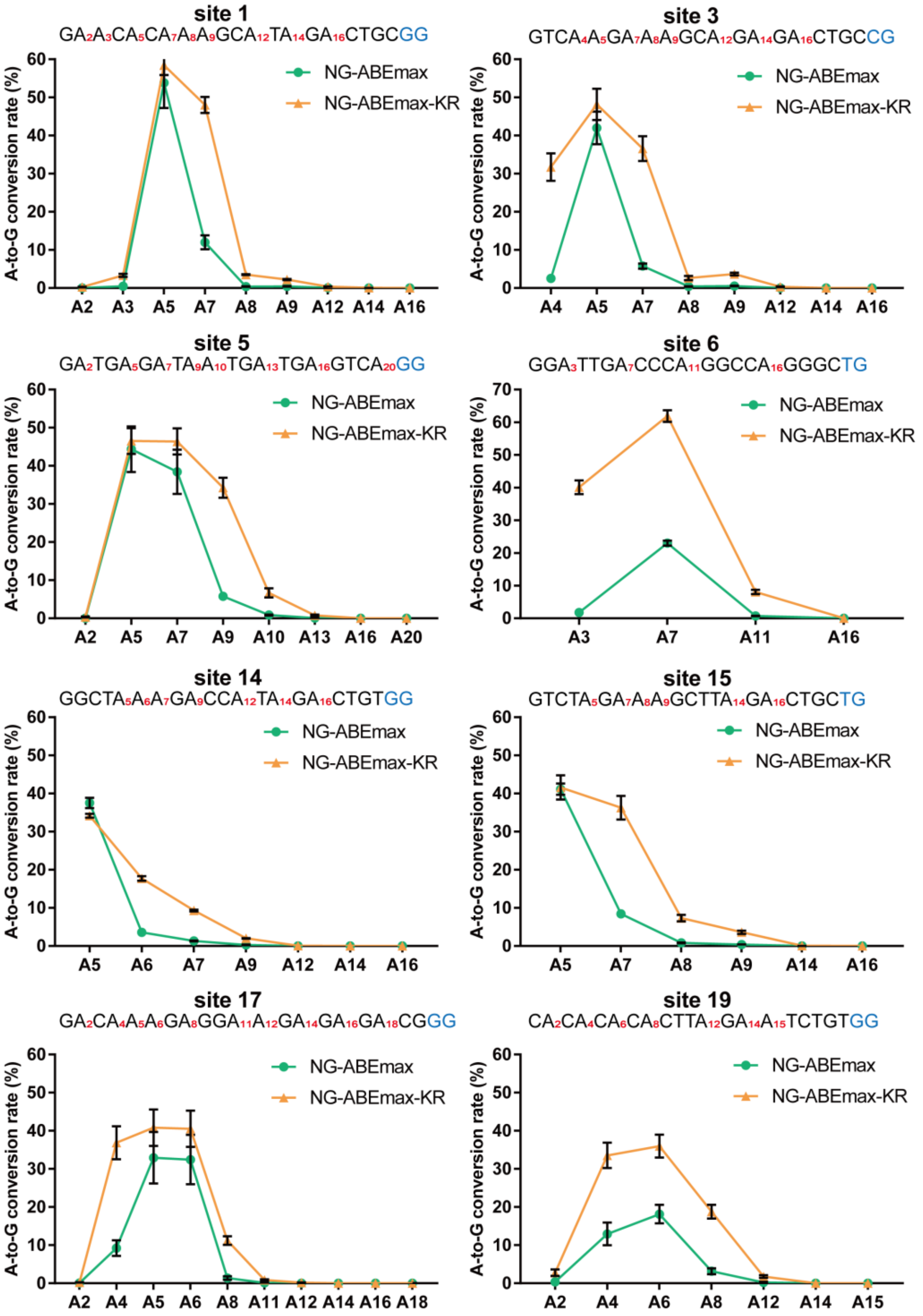

Supplementary Fig. 10 Increased editing window of NG-ABEmax-KR at endogenous genomic sites.

Summary of increased editing window of NG-ABEmax-KR in different endogenous genomic sites. Each A has been highlighted in red. PAM sequence is in blue. Error bars indicate mean ± s.d. ($n = 3$ independent experiments).

a

NG-ABEmax

	420 His	421 Asn	422 Arg	423 Asp	424 Ser	425 Tyr	426 Met	Frenquency (%)
	CCATAACAGAGACTCTTACATGG							

Mouse	Sequence	Frenquency (%)	
Tyr #1	CCATGGCAGAGACTCTTACATGG	48.61	(N421G)
	CCGTGGCAGAGACTCTTACATGG	47.65	(H420R, N421G)
Tyr #5	CCGTGACGGAGACTCTTACATGG	49.42	(H420R, N421D, R422G)
	CCATGACAGAGACTCTTACATGG	35.09	(N421D)
	CCGTGACAGAGACTCTTACATGG	11.66	(H420R, N421D)
Tyr #6	CCGTGACAGAGACTCTTACATGG	50.03	(N420R, N421D)
	CCGTGGCAGAGACTCTTACATGG	46.60	(H420R, N421G)
Tyr #7	CCATGGCAGAGACTCTTACATGG	97.25	(N421G)

b

NG-ABEmax-KR

Mouse	Sequence	Frenquency (%)	
Tyr #2	CCGTGGCGGAGACTCTTACATGG	86.2	(H420R, N421G, R422G)
	CCGTGGCAGAGACTCTTACATGG	7.05	(H420R, N421G)
Tyr #3	CCGTGGCGGAGACTCTTACATGG	72.77	(H420R, N421G, R422G)
	CCGTGGCAGAGACTCTTACATGG	20.62	(H420R, N421G)
Tyr #10	CCGTGGCGGAGACTCTTACATGG	48.32	(H420R, N421G,R422G)
	CCATGGCAGAGACTCTTACATGG	45.95	(N421G)

Supplementary Fig. 11 Sequence information of mice generated with NG-ABEmax-KR.

The *Tyr* gene information from each mouse was obtained from next-generation sequencing. The ID of mice was labeled with *Try* #. The predicted partial proteins information has been provided in the brackets. Editing information of NG-ABEmax(**a**) and NG-ABEmax-KR(**b**).

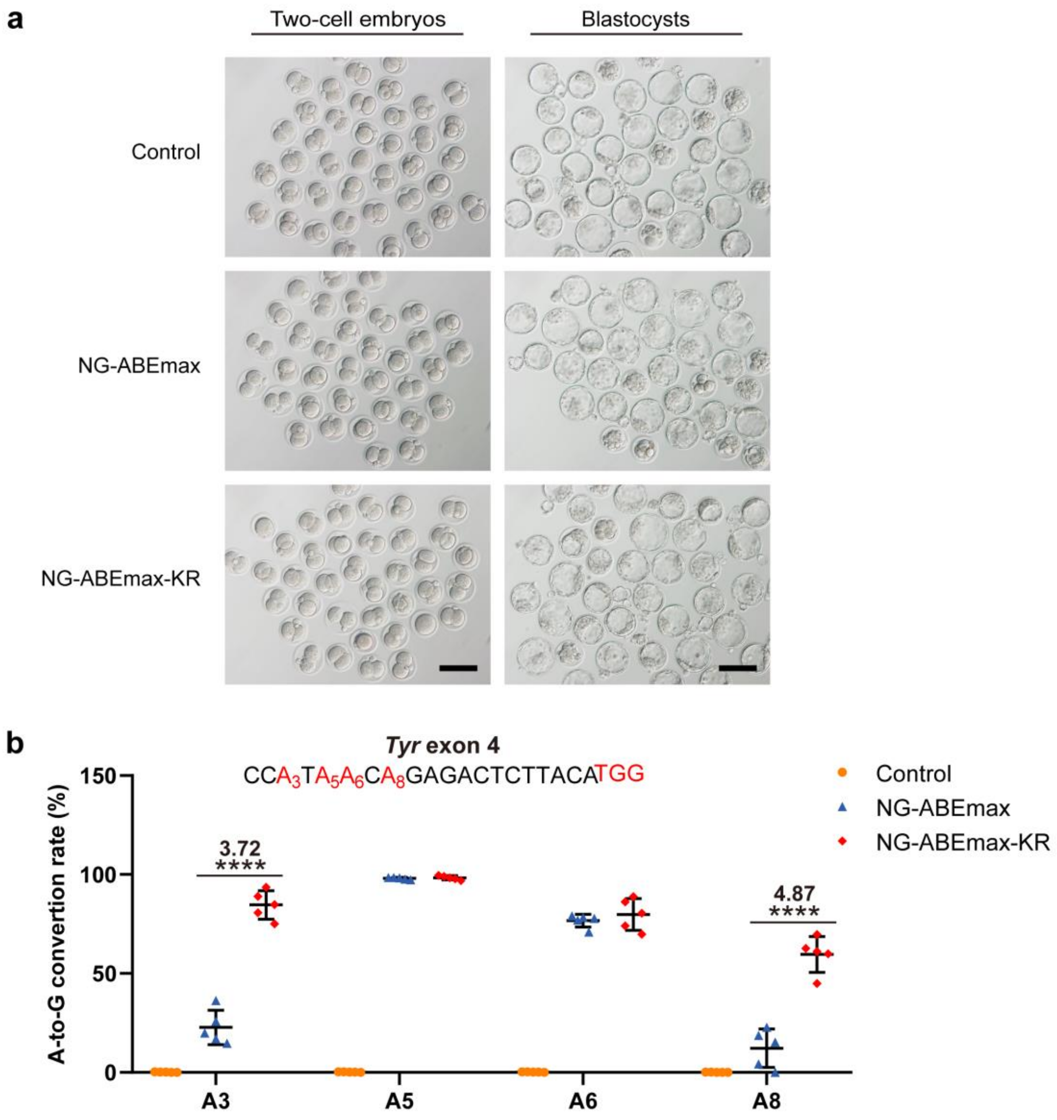

Supplementary Fig. 12 Editing efficiencies of NG-ABEmax or NG-ABEmax-KR in mouse embryos.

a Images of pre-implantation embryos obtained by injection of NG-ABEmax or NG-ABEmax-KR mRNA and sgRNA to target Tyr into zygotes. Control represents only ddH2O injection. Scale bar, 100 μm. **b** Statistical analysis of on-target A-to-G base conversions induced by NG-ABEmax or NG-ABEmaxKR in all blastocysts. Total embryos from each group were randomly divided into five groups; Data are mean ± s.d. for five groups of blastocysts. Each A base was highlighted in red. ****$P < 0.0001$ by Student's unpaired two-sided t-test. Exact P value of A3 = 0.000002, exact P value of A8 = 0.000044.

A-to-G conversion rate (%)

HEK2	G	A2	A3	C	A5	C	A7	A8	A9	G	C	A12	T	A14	G	A16	C	T	G	C
NG-ABEmax		0.04	0.43		47.98		11.11	0.48	0.44			0.08		0.03		0.01				
NG-ABEmax-KR		0.16	2.27		48.18		36.77	2.35	1.5			0.2		0.04		0.02				
NC		0.01	0.07		2.07		1.63	0.12	0.05			0.02		0.01		0.01				

A-to-G conversion rate (%)

HEK2-OT1	G	A2	A3	C	A5	C	A7	A8	T	G	C	A12	T	A14	G	A16	T	T	G	C
NG-ABEmax		0.02	0.02		0.02		0.01	0.01				0.01		0.01		0.01				
NG-ABEmax-KR		0.02	0.02		0.06		0.02	0.01				0.01		0.01		0.01				
NC		0.01	0.01		0.01		0.01	0.01				0.02		0.01		0.01				

A-to-G conversion rate (%)

HEK2-OT2	A1	A2	A3	C	A5	T	A7	A8	A9	G	C	A12	T	A14	G	A16	C	T	G	C
NG-ABEmax	0.01	0.01	0.01		2.75		0.2	0.02	0.03			0.01		0.01		0.01				
NG-ABEmax-KR	0.01	0.01	0.06		5.57		1.67	0.05	0.05			0.02		0.02		0.01				
NC	0.01	0.01	0.01		0.01		0.01	0.02	0.02			0.01		0.01		0.01				

A-to-G conversion rate (%)

HEK3	G	G	C	C	C	A6	G	A8	C	T	G	A12	G	C	A15	C	G	T	G	A20
NG-ABEmax						14.32		1.37				0.11			0.02					0.03
NG-ABEmax-KR						40.37		17.48				0.65			0.01					0.01
NC						0.02		0.02				0.03			0.01					0.02

A-to-G conversion rate (%)

HEK3-OT1	C	A2	C	C	C	A6	G	A8	C	T	G	A12	G	C	A15	C	G	T	G	C
NG-ABEmax		0.01				0.02		0.02				0.03			0.01					
NG-ABEmax-KR		0.01				0.03		0.04				0.02			0.01					
NC		0.01				0.01		0.01				0.02			0					

A-to-G conversion rate (%)

HEK3-OT2	G	A2	C	A4	C	A6	G	A8	C	C	G	G	G	C	A15	C	G	T	G	A20
NG-ABEmax		0.01		0.01		0.03		0.01							0.01					0.02
NG-ABEmax-KR		0.01		0.02		0.03		0.01							0					0.02
NC		0.01		0.01		0.02		0.01							0.01					0.02

A-to-G conversion rate (%)

HEK3-OT3	A1	G	A3	C	C	A6	G	A8	C	T	G	A12	G	C	A15	A16	G	A18	G	A20
NG-ABEmax	0.02		0.03			0.02		0.02				0.61			0.01	0.01		0.02		0.52
NG-ABEmax-KR	0.01		0.04			0.02		0.02				0.41			0.01	0.02		0.02		0.35
NC	0.01		0.03			0.02		0.02				0.19			0.02	0.01		0.02		0.14

A-to-G conversion rate (%)

HEK3-OT4	G	A2	G	C	C	A6	G	A8	A9	T	G	A12	G	C	A15	C	G	T	G	A20
NG-ABEmax		0.03				0.01		0.01	0.01			0.05			0.01					0.05
NG-ABEmax-KR		0.02				0.01		0.01	0.01			0.04			0.01					0.04
NC		0.02				0.01		0.01	0.01			0.04			0.01					0.04

A-to-G conversion rate (%)

HEK4	G	G	C	A4	C	T	G	C	G	G	C	T	G	G	A15	G	G	T	G	G
NG-ABEmax				3.76											0					
NG-ABEmax-KR				33.22											0					
NC				1.36											0					

A-to-G conversion rate (%)

HEK4-OT1	T	G	C	A4	C	T	G	C	G	G	C	C	G	G	A15	G	G	A18	G	G
NG-ABEmax				0.34								0.03						0.03		
NG-ABEmax-KR				3.63								0.03						0.03		
NC				0.06								0.02						0.02		

A-to-G conversion rate (%)

HEK4-OT2	G	G	C	T	C	T	G	C	G	G	C	T	G	G	A15	G	G	G	G	G
NG-ABEmax															0.03					
NG-ABEmax-KR															0.02					
NC															0.02					

A-to-G conversion rate (%)

HEK4-OT3	G	G	C	A4	T	C	A7	C	G	G	C	T	G	G	A15	G	G	T	G	G
NG-ABEmax				0.31			0.74								0.04					
NG-ABEmax-KR				4.69			4.36								0.04					
NC				0.01			0.06								0.03					

A-to-G conversion rate (%)

HEK4-OT4	G	G	C	G	C	T	G	C	G	G	C	G	G	G	A15	G	G	T	G	G
NG-ABEmax															0.01					
NG-ABEmax-KR															0.03					
NC															0.02					

Supplementary Fig. 13 Off-target of NG-ABEmax and NG-ABEmax-KR at endogenous genomic sites.

There are three target sites (HEK2, HEK3 and HEK4) and corresponding off-target sites (labeled with OT#) were selected for off-target test. On-target and off-target editing efficiencies are in blue and red, respectively. Median values are shown, generated from *n*=3 biological replicate.

A-to-G conversion rate (%)

Tyr exon 4	C	C	A3	T	A5	A6	C	A8	G	A10	G	A12	C	T	C	T	T	A18	C	A20
NG-ABEmax-1			48.91		99.95	99.69		0.16		0.07		0.01						0.01		0.01
NG-ABEmax-2			46.54		99.96	99.71		0.09		0.05		0.01						0.01		0.01
NG-ABEmax-3			99.02		99.02	99.02		57.84		0.98		0						0		0
NG-ABEmax-4			99.84		99.96	50.97		0.04		0.02		0.01						0.01		0.01
NG-ABEmax-5			9.65		99.82	91.38		1.25		0.07		0.01						0.01		0.01
NG-ABEmax-6			63.55		99.95	0.62		51.45		0.04		0.01						0.01		0.01
NG-ABEmax-7			49.25		99.93	5.01		0.91		4.1		0.02						0.01		0.01
NG-ABEmax-8			40.23		99.9	60.04		31.33		0.07		0.07						0.01		0.01
NG-ABEmax-9			16.34		99.92	67.29		0.53		0.04		0.01						0.01		0.01
NG-ABEmax-10			49.22		99.81	99.74		48.91		0.14		0.03						0.01		0.01
NG-ABEmax-KR-1			77.37		99.92	96.04		44.76		21.06		0						0		0
NG-ABEmax-KR-2			99.5		99.87	99.56		99.04		48.37		0.01						0.01		0.01
NG-ABEmax-KR-3			99.09		99.77	99.29		49.7		39		0.01						0.01		0.01
NG-ABEmax-KR-4			99.11		99.84	99.46		82.42		0.09		0.01						0.01		0.01
NG-ABEmax-KR-5			98.05		98.39	94.99		25.51		0.68		0.17						0		0.01
NG-ABEmax-KR-6			99.08		99.18	98.98		52.83		0.26		0.11						0.01		0.01
NG-ABEmax-KR-7			99.15		99.29	99.16		98.64		0.45		0.12						0.01		0.01
NG-ABEmax-KR-8			99.17		99.31	99.15		75.3		0.35		0.08						0.01		0.01
NG-ABEmax-KR-9			25.71		99.02	98.88		98.55		0.44		0.17						0.01		0.01
NG-ABEmax-KR-10			77.94		80.48	80.4		80.11		0.69		0.12						0.01		0.01

A-to-G conversion rate (%)

Off target-1	T	T	A3	T	A5	A6	C	A8	G	A10	G	A12	C	T	C	T	T	A18	C	T
NG-ABEmax-1			0.01		0.01	0.01		0.01		0.01		0.01						0.02		
NG-ABEmax-2			0.01		0.01	0.01		0.01		0.01		0.01						0.03		
NG-ABEmax-3			0.01		0.01	0.01		0.01		0.01		0.01						0.02		
NG-ABEmax-4			0		0.01	0.01		0.01		0.01		0.01						0.02		
NG-ABEmax-5			0.01		41.82	41.84		41.83		0.01		0.01						0.02		
NG-ABEmax-6			0		0.06	0.06		0.05		0.01		0.01						0.02		
NG-ABEmax-7			0.01		0.01	0.02		0.01		0.01		0.01						0.03		
NG-ABEmax-8			0		0.01	0.01		0.01		0.02		0.01						0.03		
NG-ABEmax-9			20.2		51.92	0.03		72.2		0.02		0.01						0.02		
NG-ABEmax-10			28.19		28.32	0.05		28.34		0.01		0.02						0.03		
NG-ABEmax-KR-1			0		0.13	0.01		0.07		0.01		0.01						0.02		
NG-ABEmax-KR-2			0.01		38.21	0.02		38.07		0.01		0.01						0.02		
NG-ABEmax-KR-3			0.01		42.05	0.02		0.05		0.01		0.01						0.02		
NG-ABEmax-KR-4			0		0.01	0.01		0.01		0.01		0.01						0.02		
NG-ABEmax-KR-5			0.01		0.02	0.02		0.02		0.01		0						0.02		
NG-ABEmax-KR-6			0.01		0.01	0.01		0.01		0.01		0.01						0.01		
NG-ABEmax-KR-7			0.01		0.01	4.15		0.01		0.01		0.01						0.02		
NG-ABEmax-KR-8			0.01		0.01	0.01		0.01		0.01		0.01						0.02		
NG-ABEmax-KR-9			0.01		0.02	0.01		39.08		0.01		0.01						0.01		
NG-ABEmax-KR-10			0.01		0.01	0.01		0.02		0		0.01						0.01		

A-to-G conversion rate (%)

Off target-2	C	G	A3	T	T	A6	C	A8	G	A10	G	A12	C	T	C	C	T	A18	C	A20
NG-ABEmax-1			0.01			0.01		0.02		0.01		0.01						0.01		0.01
NG-ABEmax-2			0.01			0.01		0.02		0.01		0.02						0.01		0.01
NG-ABEmax-3			0.01			0.01		0.02		0.01		0.01						0.01		0.01
NG-ABEmax-4			0			0.01		0.02		0.01		0.01						0.01		0.01
NG-ABEmax-5			0.01			0.01		0.02		0.02		0.01						0.01		0.01
NG-ABEmax-6			0			0.01		0.01		0.01		0.01						0.01		0.02
NG-ABEmax-7			0			0.01		0.02		0.01		0.01						0		0
NG-ABEmax-8			0.01			0		0.02		0		0.01						0.01		0.01
NG-ABEmax-9			0.01			0.01		0.01		0.01		0.01						0.01		0.01
NG-ABEmax-10			0.01			0.01		0.01		0.01		0.01						0.01		0.01
NG-ABEmax-KR-1			0.01			0.01		0.02		0.02		0.01						0.01		0.01
NG-ABEmax-KR-2			0.01			0.01		0.02		0.02		0.01						0.01		0.01
NG-ABEmax-KR-3			0			0.01		0.02		0.01		0.01						0.01		0.01
NG-ABEmax-KR-4			0			0.01		0.02		0.02		0.01						0.01		0.01
NG-ABEmax-KR-5			0			0.01		0.01		0.01		0.01						0		0.01
NG-ABEmax-KR-6			0			0.01		0.02		0.01		0.01						0.01		0.01
NG-ABEmax-KR-7			0			0.01		0.02		0.01		0.01						0		0.01
NG-ABEmax-KR-8			0.01			0.01		0.01		0.01		0.01						0.01		0.01
NG-ABEmax-KR-9			0			0.01		0.01		0.01		0.01						0.01		0.01
NG-ABEmax-KR-10			0.01			0.01		0.01		0.01		0.01						0		0.01

A-to-G conversion rate (%)

Off target-3	C	C	A3	T	A5	A6	C	T	G	A10	G	A12	C	T	C	T	G	C	C	A20
NG-ABEmax-1			0.01		0.01	0.01				0.01		0.02								0.01
NG-ABEmax-2			0.01		0.01	0.01				0.02		0.01								0.01
NG-ABEmax-3			0.01		0.01	0.01				0.02		0.01								0.01
NG-ABEmax-4			0.01		0.01	0.01				0.01		0.01								0.01
NG-ABEmax-5			0.01		0.01	0.01				0.01		0.01								0.01
NG-ABEmax-6			0		0.01	0.01				0.01		0.01								0.01
NG-ABEmax-7			0.01		0.01	0.02				0.02		0.02								0.02
NG-ABEmax-8			0.01		0	0.01				0.01		0.01								0.01
NG-ABEmax-9			0.01		0.01	0.01				0.02		0.01								0.01
NG-ABEmax-10			0.01		0	0.01				0.01		0.01								0.01
NG-ABEmax-KR-1			0.01		0	0.01				0.01		0.01								0.01
NG-ABEmax-KR-2			0.01		0.01	0.01				0.01		0.01								0.01
NG-ABEmax-KR-3			0.01		0.01	0				0.02		0.02								0.01
NG-ABEmax-KR-4			0.01		0.01	0.01				0.01		0.01								0.01
NG-ABEmax-KR-5			0.01		0.01	0.01				0.01		0.01								0.01
NG-ABEmax-KR-6			0.01		0.01	0.01				0.01		0.01								0.01
NG-ABEmax-KR-7			0.01		0.01	0.01				0.01		0.01								0.01
NG-ABEmax-KR-8			0.01		0.01	0.01				0.01		0.01								0.01
NG-ABEmax-KR-9			0.01		0.01	0.01				0.01		0.01								0.01
NG-ABEmax-KR-10			0.01		0.01	0.01				0.01		0.01								0.01

A-to-G conversion rate (%)

Off target-4	C	A2	A3	T	A5	A6	C	A8	G	A10	G	G	T	T	C	T	T	A18	C	A20
NG-ABEmax-1		0	0		0	0		0		0								0		0
NG-ABEmax-2		0.01	0.01		0.01	0.01		0.01		0.01								0.01		0.01
NG-ABEmax-3		0.01	0		0.01	0		0.01		0.01								0.01		0.02
NG-ABEmax-4		0.01	0.01		0.01	0.01		0.01		0.01								0.01		0.01
NG-ABEmax-5		0.01	0.01		0.01	0		0.01		0.01								0.01		0.01
NG-ABEmax-6		0	0.01		0	0		0.02		0.01								0.01		0.02
NG-ABEmax-7		0	0.01		0.01	0.01		0.01		0.01								0.01		0.02
NG-ABEmax-8		0.01	0.01		0.01	0		0.01		0.01								0.01		0.01
NG-ABEmax-9		0.02	0.01		0	0		0.01		0.01								0		0.01
NG-ABEmax-10		0.01	0.01		0.01	0.01		0.02		0.01								0.01		0.02
NG-ABEmax-KR-1		0.01	0.01		0.01	0		0.01		0.01								0.01		0.01
NG-ABEmax-KR-2		0.01	0.01		0.01	0.01		0.01		0.01								0.01		0.01
NG-ABEmax-KR-3		0.01	0.01		0.01	0.01		0.01		0.02								0.01		0.01
NG-ABEmax-KR-4		0.01	0.01		0.01	0.01		0.01		0.01								0.01		0.01
NG-ABEmax-KR-5		0.01	0.01		0.01	0		0.01		0.01								0.01		0.01
NG-ABEmax-KR-6		0.01	0.01		0.01	0.01		0.01		0.01								0.01		0.01
NG-ABEmax-KR-7		0.01	0.01		0.01	0.01		0.01		0.01								0.01		0.01
NG-ABEmax-KR-8		0.01	0.01		0	0		0.01		0.01								0.01		0.01
NG-ABEmax-KR-9		0.01	0.01		0.01	0		0.01		0.01								0.01		0.01
NG-ABEmax-KR-10		0.01	0.01		0.01	0.01		0.01		0.01								0		0.01

A-to-G conversion rate (%)

Off target-5	A1	C	A3	T	A5	G	C	A8	G	G	G	A12	C	T	C	T	T	A18	C	A20
NG-ABEmax-1	0.01		0.01		0.01			0.01				0.01						0.01		0.02
NG-ABEmax-2	0.01		0.01		0.01			0.01				0.01						0.01		0.02
NG-ABEmax-3	0.01		0.01		0.02			0.02				0.01						0.01		0.01
NG-ABEmax-4	0.01		0.01		0.06			0.06				0.01						0.02		0.02
NG-ABEmax-5	0.01		0.01		0.01			0.01				0.01						0.01		0.02
NG-ABEmax-6	0.01		0.01		0.01			0.01				0.01						0.02		0.03
NG-ABEmax-7	0.01		0.01		0.01			0.02				0.01						0.01		0.02
NG-ABEmax-8	0.02		0.01		0.02			0.01				0.01						0.01		0.02
NG-ABEmax-9	0.01		0.01		0.01			0.01				0.01						0.02		0.02
NG-ABEmax-10	0.02		0.01		0.02			0.02				0.01						0.01		0.02
NG-ABEmax-KR-1	0.01		0.01		46.4			46.4				0.01						0.01		0.03
NG-ABEmax-KR-2	0.01		0.01		0.02			0.02				0.01						0.01		0.02
NG-ABEmax-KR-3	0.02		0.01		0.02			0.03				0.01						0.01		0.02
NG-ABEmax-KR-4	0.01		0.01		0.01			0.01				0.01						0.01		0.02
NG-ABEmax-KR-5	0.02		0.03		13.07			0.02				0.01						0.02		0.02
NG-ABEmax-KR-6	0.01		0.01		0.01			0.06				0.01						0.01		0.02
NG-ABEmax-KR-7	0.02		0		0.06			0.02				0.01						0.01		0.03
NG-ABEmax-KR-8	0.01		0.01		25.64			0.02				0.01						0.01		0.02
NG-ABEmax-KR-9	0.03		0.01		0.01			0.02				0.01						0.01		0.02
NG-ABEmax-KR-10	25.81		0.01		25.85			25.81				0.01						0.02		0.02

A-to-G conversion rate (%)

Off target-6	T	C	C	A4	A5	A6	C	A8	G	A10	G	A12	C	T	C	T	T	A18	C	A20
NG-ABEmax-1				0.01	0.01	0		0.01		0.01		0.01						0.01		0.01
NG-ABEmax-2				0.01	0.01	0.01		0.01		0.01		0.01						0.01		0.02
NG-ABEmax-3				0.01	0.01	0		0.01		0.01		0.01						0.01		0.02
NG-ABEmax-4				0.01	0.01	0.01		0		0.01		0.02						0.01		0.01
NG-ABEmax-5				0.01	0.01	0.01		0.01		0.01		0.01						0.01		0.01
NG-ABEmax-6				0.01	0.02	0		0.01		0.01		0.01						0.02		0.03
NG-ABEmax-7				0.01	0.01	0.01		0.01		0.01		0.01						0.01		0.01
NG-ABEmax-8				0.01	0.01	0		0.01		0.01		0.01						0.01		0.02
NG-ABEmax-9				0.01	0.01	0.01		0.01		0.01		0.01						0.01		0.01
NG-ABEmax-10				0.01	0.01	0.01		31.56		0.01		0.01						0.01		0.02
NG-ABEmax-KR-1				0.01	0.01	0.01		0.01		0.01		0.01						0.01		0.02
NG-ABEmax-KR-2				0.01	0.01	0.01		0.01		0.02		0.01						0.01		0.01
NG-ABEmax-KR-3				0.01	0.02	0.01		0.05		0.02		0.01						0.01		0.02
NG-ABEmax-KR-4				0.01	0.01	3.61		30.05		0.02		0.02						0.01		0.02
NG-ABEmax-KR-5				0.01	0.01	0		0.01		0.01		0.01						0.01		0.01
NG-ABEmax-KR-6				0.01	0.01	0.01		0.01		0.01		0.01						0.01		0.01
NG-ABEmax-KR-7				0.01	0.01	0.01		0.01		0.01		0.01						0.01		0.02
NG-ABEmax-KR-8				0.01	0.01	0.01		0		0.01		0.01						0.01		0.02
NG-ABEmax-KR-9				0.01	0.01	0.01		0.01		0.01		0.01						0.01		0.01
NG-ABEmax-KR-10				0.01	0.01	0.01		0.02		0.01		0.01						0.01		0.01

Supplementary Fig. 14 Off-target of NG-ABEmax and NG-ABEmax-KR at mouse *Try* gene.

Four mice harboring the edited A3 base from each group have been selected. Six off-target sites (labeled with Off target-#) were selected for off-target test. Off-target editing efficiencies are in red.

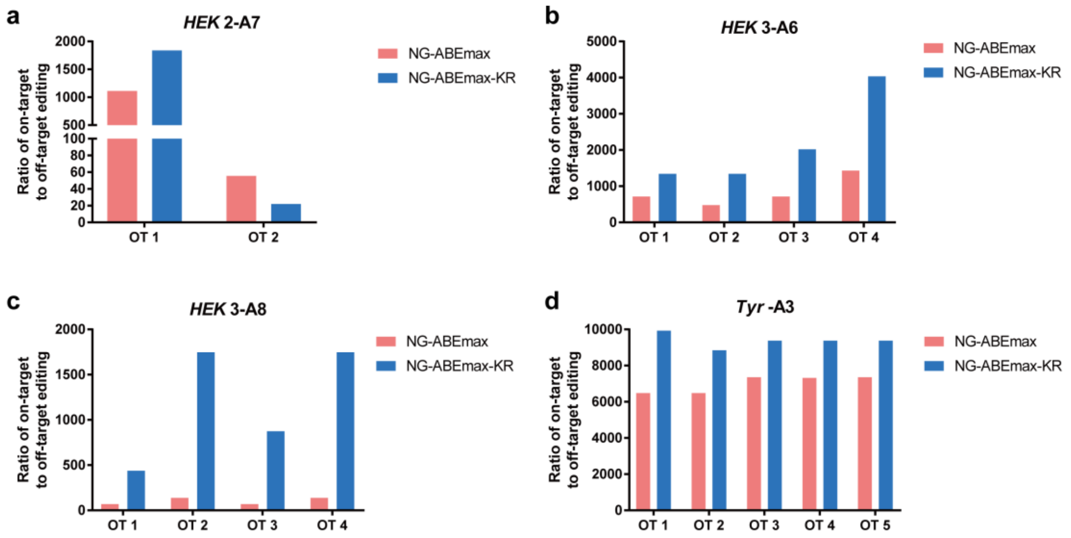

Supplementary Fig. 15 Higher ratio of on-target to off-target editing of NG-ABEmax-KR at human endogenous gene and mouse *Try* gene.

On-target and off-target editing efficiency of NG-ABEmax and NG-ABEmax-KR at different sites were performed. Data from OT site represent the ratio of on-target to off-target editing. The data of sites from human endogenous genes (*HEK* 2, *HEK* 3) are showed (**a, b** and **c**). The *Try* on-target and off-target editing ratio from four mice are summarized (**d**).

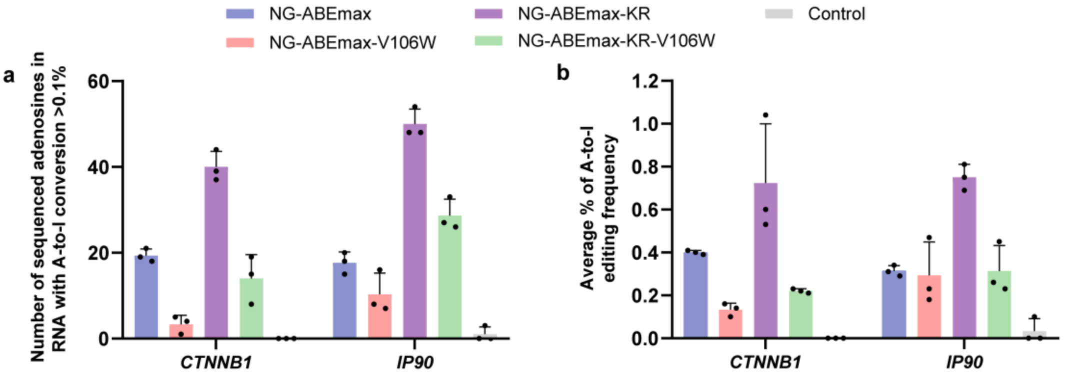

Supplementary Fig. 16 RNA off-target editing of NG-ABEmax-KR and variants with two mutations (E59A in TadA, V106W in TadA*).

a The number of adenosines converted to inosine at a detectable level (>0.1%) of the corresponding mRNA. b. Average A-to-I RNA editing frequencies by NG-ABEmax, NG-ABEmax-KR and variants with AW (E59A in TadA, V106W in TadA*). Error bars indicate mean ± s.d ($n = 3$ independent experiments).

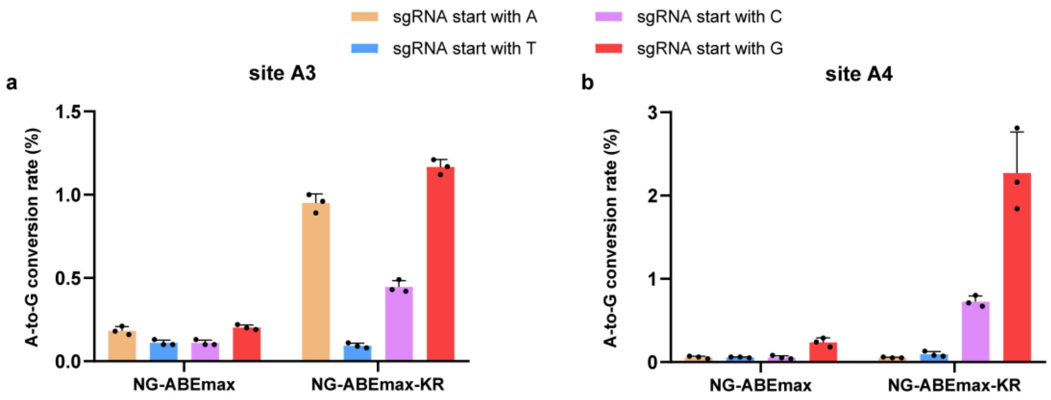

Supplementary Fig. 17 Base editing efficiencies for sgRNA started with different first nucleotide.

a The editing efficiencies of NG-ABEmax and NG-ABEmax-KR with four sgRNA targeted at position A3 in the EGFP (Figure 1d) in HEK-293 cells. **b** The editing efficiencies at position A4 in HEK-293 cells. Error bars indicate mean ± s.d (n = 3 independent experiments).

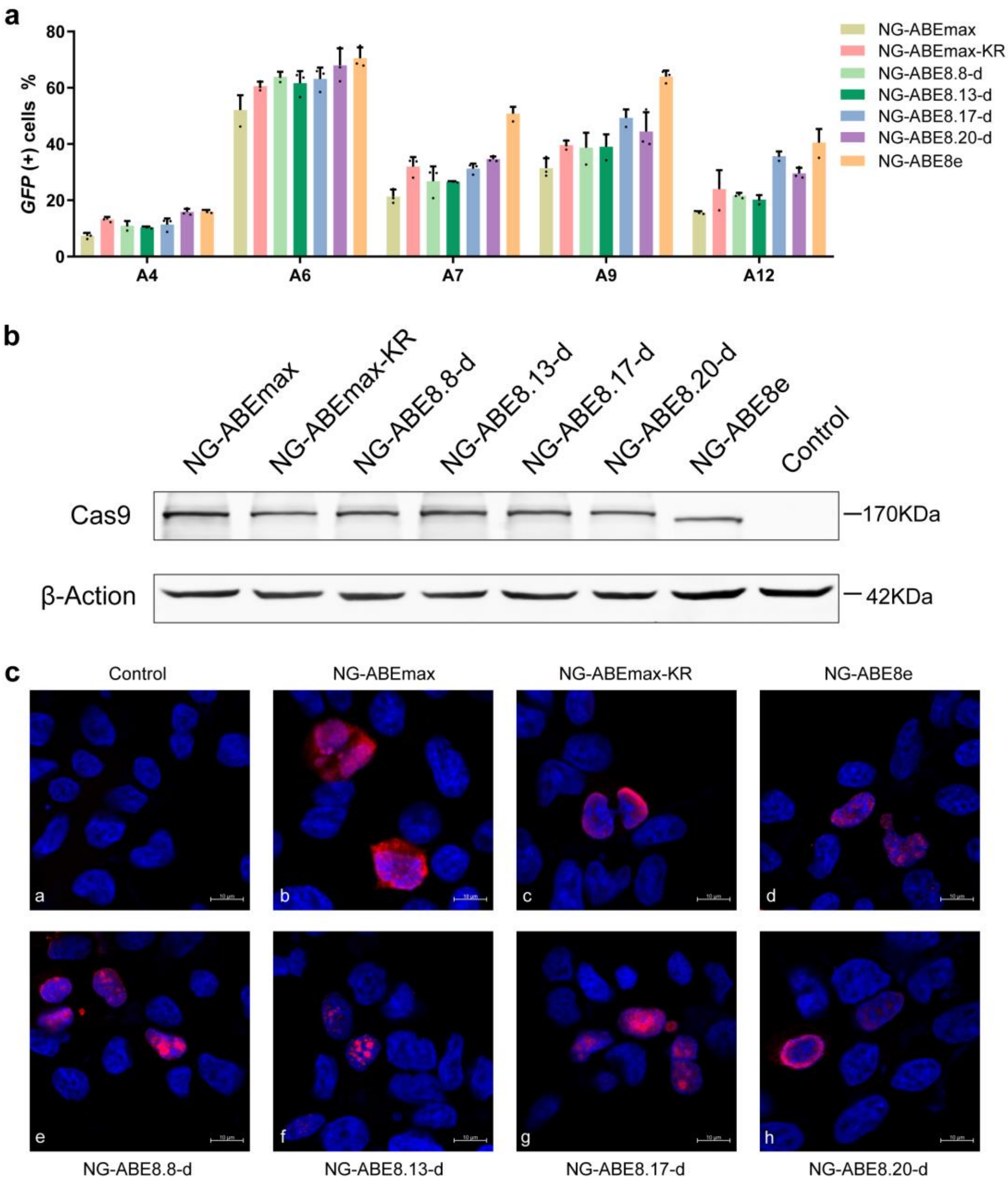

Supplementary Fig. 18 Activity, protein expression levels and nuclear localization of ABE variants.

a Base editing induced by ABEs at different A base in EGFP. Error bars indicate mean ± s.d. (*n* = 3 independent experiments). **b** Western blot analysis of ABE variants via the recognition with specific Cas9 antibody. **c** Nuclear localization of ABE variants. HEK-293 cells were transfected with the plasmids, fixed 24 hours post-transfection and stained with the antibody against Cas9 (red). DAPI (blue) indicated the nucleus. Scale bar is 10 µm.

Supplementary Table. 1 Mutations information of randomly picked variants.

Colony ID	Mutation number/575 bp	Colony ID	Mutation number /730 bp
S-L1-1	2	S-L2-1	1
S-L1-2	3	S-L2-2	6
S-L1-3	Deletion	S-L2-3	4
S-L1-4	1	S-L2-4	2
S-L1-5	2	S-L2-5	5
S-L1-6	5	S-L2-6	6
S-L1-7	4	S-L2-7	2
S-L1-8	1	S-L2-8	1
S-L1-9	2	S-L2-9	3
S-L1-10	4	S-L2-10	3
S-L1-11	1	S-L2-11	1
S-L1-12	1	S-L2-12	3
S-L1-13	3	S-L2-13	3
S-L1-14	3	S-L2-14	3
S-L1-15	1	S-L2-15	6
S-L1-16	5	S-L2-16	Deletion
S-L1-17	5	S-L2-17	8
S-L1-18	5	S-L2-18	1
S-L1-19	1	S-L2-19	6
S-L1-20	5	S-L2-20	3

Supplementary Table. 2 Summary of mutations information.

NG-ABEmax variants		Mut1	Mut2	Mut3	Mut4	Mut5	Mut6	Mut7	Mut8
L1-42	evolved TadA	L145L (c.1083G>A)	Q154R						
L1-50		R101R (c.951C>G)	E134D						
L1-60		R101S	D139G	E140K					
L1-66		E3A	M61L	T79S	T83T (c.897A>G)	S97S (c.939T>A)	V102V (c.954G>A)	M126L	M151L
L1-79		N37S	R98R (c.942G>A)	T133S	Q154R				
L1-98		E9K	Q154R						
L1-240		E43V	W45R	I136I (c.1056C>T)	M151L	D167E			
L1-244		N127K							
L2-106	wild-type TadA	R150G							
L2-151		V33M	Q71Ter	N127K					
L2-156		A106S	N127K	E155K					
L2-159		Y81Y (c.297T>C)	Q159H						

Supplementary Table. 3 No toxic effect of NG-ABEmax-KR for mouse development.

Methods	No. of injected embryos	No. of two-cell embryos (%)	No. of blastocysts (%)	No. of transferred embryos	No. of offsprings (%)
H_2O injection	40	39 (97.5)[a]	30 (76.9)[b]	NA	NA
NG-ABEmax mRNA+ Tyr sgRNA	36	35 (97.2)[a]	26 (74.3)[b]	NA	NA
NG-ABEmax-KR mRNA+ Tyr sgRNA	39	37 (94.9)[a]	34 (91.9)[b]	NA	NA
H_2O injection	56	54 (96.4)[a]	NA	54	21 (38.9)[c]
NG-ABEmax mRNA+ Tyr sgRNA	32	30 (93.8)[a]	NA	30	12 (40)[c]
NG-ABEmax-KR mRNA+ Tyr sgRNA	63	60 (95.2)[a]	NA	60	22 (36.7)[c]

NA: not applicable.

[a]Calculated from the number of injected embryos.

[b]Calculated from the number of developed two-cell stage embryos.

[c]Calculated from the number of transferred embryos.

Supplementary Table. 4 Primers used to generate different *EGFP* variants.

Primer Description	Primer sequence (5'-3')
fwd_EGFP	CAGGTGTCGTGAGGGAATTCGATTATCGCC
rev_dEGFP1	CGTAGGTCAGCGACGACACCAGCGAGGGCTAGGGCA
fwd_dEGFP1	TAGCCCTCGCTGGTGTCGTCGCTGACCTACGG
rev_dEGFP2	CGACACCAGCGAGGGTCAGGGCACGGG
fwd_dEGFP2	CCCTGACCCTCGCTGGTGTCGTCGCTGA
rev_EGFP-variant	GTAGGTCAGCGACGACACCAGCGAGGGCCAGGGCA
fwd_EGFP-variant	TGGCCCTCGCTGGTGTCGTCGCTGACCTACGG
rev_EGFP	CCCTAGATGCATGCGGATCCTTCGAACTAG

Supplementary Table. 5 Primers used to generate sgRNA plasmids targeting different sites of *dEGFP1* or *dEGFP2*.

Primer Description	Primer sequence (5'-3')
dEGFP1-A3 F	CACCCTAGCCCTCGCTGGTGTCGT
dEGFP1-A3 R	AAACACGACACCAGCGAGGGCTAG
dEGFP1-A6 F	CACCGCCCTAGCCCTCGCTGGTGT
dEGFP1-A6 R	AAACACACCAGCGAGGGCTAGGGC
dEGFP1-A9 F	CACCCGTGCCCTAGCCCTCGCTGG
dEGFP1-A9 R	AAACCCAGCGAGGGCTAGGGCACG
dEGFP1-A11 F	CACCCCCGTGCCCTAGCCCTCGCT
dEGFP1-A11 R	AAACAGCGAGGGCTAGGGCACGGG
dEGFP1-A12 F	CACCGCCCGTGCCCTAGCCCTCGC
dEGFP1-A12 R	AAACGCGAGGGCTAGGGCACGGGC
dEGFP1-A15 F	CACCGCTGCCCGTGCCCTAGCCCT
dEGFP1-A15 R	AAACAGGGCTAGGGCACGGGCAGC
dEGFP2-A1 F	CACCACCCTCGCTGGTGTCGTCGC
dEGFP2-A1 R	AAACGCGACGACACCAGCGAGGGT
dEGFP2-A4 F	CACCCTGACCCTCGCTGGTGTCGT
dEGFP2-A4 R	AAACACGACACCAGCGAGGGTCAG
dEGFP2-A7 F	CACCGCCCTGACCCTCGCTGGTGT
dEGFP2-A7 R	AAACACACCAGCGAGGGTCAGGGC
dEGFP2-A10 F	CACCCGTGCCCTGACCCTCGCTGG
dEGFP2-A10 R	AAACCCAGCGAGGGTCAGGGCACG
dEGFP2-A13 F	CACCGCCCGTGCCCTGACCCTCGC
dEGFP2-A13 R	AAACGCGAGGGTCAGGGCACGGGC
dEGFP2-A16 F	CACCGCTGCCCGTGCCCTGACCCT
dEGFP2-A16 R	AAACAGGGTCAGGGCACGGGCAGC

Supplementary Table. 6 Primers used to generate sgRNA plasmid targeting MCS site in the HEK293-PME cell line.

Primer Description	Primer sequence (5'-3')
MCS F	CACCTTCTGCAGTCGACGGTACCG
MCS R	AAACCGGTACCGTCGACTGCAGAA

Supplementary Table. 7 Primers used to generate sgRNA plasmids targeting different genomic loci.

Primer Description	Primer sequence (5'-3')
site1 (HEK2) F	CACCGAACACAAAGCATAGACTGC
site1 (HEK2) R	AAACGCAGTCTATGCTTTGTGTTC
site2 F	CACCGAGTATGAGGCATAGACTGC
site2 R	AAACGCAGTCTATGCCTCATACTC
site3 F	CACCGTCAAGAAAGCAGAGACTGC
site3 R	AAACGCAGTCTCTGCTTTCTTGAC
site5 F	CACCGATGAGATAATGATGAGTCA
site5 R	AAACTGACTCATCATTATCTCATC
site6 F	CACCGGATTGACCCAGGCCAGGGC
site6 R	AAACGCCCTGGCCTGGGTCAATCC
site7 F	CACCGAATACTAAGCATAGACTCC
site7 R	AAACGGAGTCTATGCTTAGTATTC
site8 F	CACCGTAAACAAAGCATAGACTGA
site8 R	AAACTCAGTCTATGCTTTGTTTAC
site9 F	CACCGAAGACCAAGGATAGACTGC
site9 R	AAACGCAGTCTATCCTTGGTCTTC
site10 F	CACCGAACATAAAGAATAGAATGA
site10 R	AAACTCATTCTATTCTTTATGTTC
site12 F	CACCGTAGAAAAAGTATAGACTGC
site12 R	AAACGCAGTCTATACTTTTTCTAC
site13 F	CACCGAAGATAGAGAATAGACTGC
site13 R	AAACGCAGTCTATTCTCTATCTTC
site14 F	CACCGGCTAAAGACCATAGACTGT
site14 R	AAACACAGTCTATGGTCTTTAGCC
site15 F	CACCGTCTAGAAAGCTTAGACTGC
site15 R	AAACGCAGTCTAAGCTTTCTAGAC
site17 F	CACCGACAAAGAGGAAGAGAGACG
site17 R	AAACCGTCTCTCTTCCTCTTTGTC
site18 F	CACCACACACACACTTAGAATCTG
site18 R	AAACCAGATTCTAAGTGTGTGTGT
HEK3 F	CACCGGCCCAGACTGAGCACGTGA
HEK3 R	AAACTCACGTGCTCAGTCTGGGCC
HEK4 F	CACCGGCACTGCGGCTGGAGGTGG
HEK4 R	AAACCCACCTCCAGCCGCAGTGCC
HBG1/2 F	CACCGTGGGGAAGGGGCCCCCAAG
HBG1/2 R	AAACCTTGGGGGCCCCTTCCCCAC
Tyr F	CACCCCATAACAGAGACTCTTACA
Tyr R	AAACTGTAAGAGTCTCTGTTATGG

Supplementary Table. 8 Primers used to identify key mutations for L1-60, L1-98 and L2-156.

Primer Description	Primer sequence (5'-3')
fwd_BamHI	TCTGGAGGATCTAGCGGAGGATCC
rev_60-1	CAAACACCACGCTGCCGATC
fwd_60-1	GGCAGCGTGGTGTTTGGCGT
rev_60-2	CGGCACATTCACCTGCCAGG
fwd_60-2	GCAGGTGAATGTGCCGCCCT
rev_60-3	CGGCACATTTATCTGCCAGG
fwd_60-3	GCAGATAAATGTGCCGCCCT
rev_60-4	CAAACACCACGCGGCCGATC
fwd_60-4	GGCCGCGTGGTGTTTGGCGT
rev_98-1	TCAGGGCATGTCTCATCCAGTACTTGTGG
fwd_98-1	CCACAAGTACTGGATGAGACATGC
rev_98-2	GCATTGAACACCCGTCTAGGCA
fwd_98-2	TAGACGGGTGTTCAATGCTCAGA
rev_ABEmax	CCTTGGCCATCTCGTTGCTGAAGATCT
fwd_TadA-mut	TCAGATCCGCTAGAGATCCGCGG
rev_W156-1	CCGTGATCCGAACACCACTCT
fwd_W156-1	GTGGTGTTCGGATCACGGGAC
rev_W156-2	GGTGCTTCATGCCGGGGTGGT
fwd_W156-2	ACCCCGGCATGAAGCACCGG
rev_W156-3	TGATCTTCTGTCTCCGCATTC
fwd_W156-3	TGCGGAGACAGAAGATCAAGG
rev_W156-4	TGATCTCCTGTCTCCGCATTC
fwd_W156-4	TGCGGAGACAGGAGATCAAGG
rev_W156-5	TGGTTCATGCCGGGGTGGTGC
fwd_W156-5	CCACCCCGGCATGAACCACC
rev_W156-6	CCGTGCTCCGAACACCACTCT
fwd_W156-6	GTGGTGTTCGGAGCACGGGAC
rev_TadA-mut	TGGGAAAACTCCACCTCAGAGGATCC

Supplementary Table. 9 Primers used to generate NG-ABEmax-R^{127} and NG-ABEmax H^{127}.

Primer Description	Primer sequence (5'-3')
fwd_TadA-mut	TCAGATCCGCTAGAGATCCGCGG
rev_ N127R	GGTGCCTCATGCCGGGGTGGT
fwd_ N127R	ACCCCGGCATGAGGCACCGG
rev_ N127H	GGTGGTGCATGCCGGGGTGGT
fwd_ N127H	ACCCCGGCATGCACCACCGGGTGG
rev_TadA-mut	TGGGAAAACTCCACCTCAGAGGATCC

Supplementary Table. 10 Primers used to generate 2 NG-ABEmax variants libraries.

Primer Description	Primer sequence (5'-3')
fwd_TadA*-mut	TCCGCCACACCAGAGAGCTCCG
rev_TadA*-mut	TCAGAGCCAGAGGAGCCTCCGCTAGAT
fwd_TadA-mut	TCAGATCCGCTAGAGATCCGCGG
rev_TadA-mut	TGGGAAAACTCCACCTCAGAGGATCC

Supplementary Table. 11 Primers used for fragments amplification.

Primer Description	Primer sequence (5'-3')
fwd_MCS	CAGGGAGCTCAAAATGGAGG
rev_MCS	TGCAGATGAACTTCAGGGTC
fwd_site1 (HEK2)	GGAGTGAGTACGGTGTGCAGACCTGGCTGAGCTAACTG
rev_site1 (HEK2)	GAGTTGGATGCTGGATGGCCCATCTGTCAAACTGTGCG
fwd_site2	GGAGTGAGTACGGTGTGCGCTTTTCAGCTAGAGGGAACCT
rev_site2	GAGTTGGATGCTGGATGGCTGAAATGCTGTGCGTGTCT
fwd_site3	GGAGTGAGTACGGTGTGCGGTTTCCTGTCTTCCCTTTCCC
rev_site3	GAGTTGGATGCTGGATGGCCGACAGCCAGTGGTTAAGT
fwd_site5	GGAGTGAGTACGGTGTGCAAGGAACTCAGGTCCAGGGAA
rev_site5	GAGTTGGATGCTGGATGGTGAGAGCAGGGACCACATCTA
fwd_site6	GGAGTGAGTACGGTGTGCGCATGCATTTGTAGGCTTGATGC
rev_site6	GAGTTGGATGCTGGATGGAGGAAAAGCTGTCCTGCGAC
fwd_site7	GGAGTGAGTACGGTGTGCTCCACCGCCTTGTTTACTGCT
rev_site7	GAGTTGGATGCTGGATGGTAGGTTTGCATAGACCTGCCC
fwd_site8	GGAGTGAGTACGGTGTGCCCCTGTTCCTAAAGCCCACC
rev_site8	GAGTTGGATGCTGGATGGACTGGTTCTGTTTGTGGCCA
fwd_site9	GGAGTGAGTACGGTGTGCGGCCCAATGACACCACATGA
rev_site9	GAGTTGGATGCTGGATGGGGAACAGGCAGCGTATTGCT
fwd_site10	GGAGTGAGTACGGTGTGCTCCACCTCCCCACTTCTCTT
rev_site10	GAGTTGGATGCTGGATGGGGTGAAATGAGCAAGGCACA
fwd_site12	GGAGTGAGTACGGTGTGCCAGGTGATGGAAAGCATTTGCC
rev_site12	GAGTTGGATGCTGGATGGACCCATGTGCCTGACATAGG
fwd_site13	GGAGTGAGTACGGTGTGCCTTCTCTTTCTCTCCCCCACCC
rev_site13	GAGTTGGATGCTGGATGGTCACTTCAGCCCAGGAGTAT
fwd_site14	GGAGTGAGTACGGTGTGCTCTAACCTGTGTGACACTTGGCA
rev_site14	GAGTTGGATGCTGGATGGGAACCTGAAGCCTTTCCCCA
fwd_site15	GGAGTGAGTACGGTGTGCAACTAGGTGGCTTCAGAGGC
rev_site15	GAGTTGGATGCTGGATGGGCACTCAGCTAGACTTAACTCCC
fwd_site17	GGAGTGAGTACGGTGTGCCCTGATTCCAAGGAGATTGCCA
rev_site17	GAGTTGGATGCTGGATGGTCCCTCCTCTGCGTGAATTTG
fwd_site18	GGAGTGAGTACGGTGTGCGCATTACCTGGGAGCCTGTT
rev_site18	GAGTTGGATGCTGGATGGAACTTCAGCGGGCATCAGAA
fwd_HEK2-off1	GGAGTGAGTACGGTGTGCGTCCAGATAATCAGAGTGTGG
rev_HEK2-off1	GAGTTGGATGCTGGATGGCTTGTGAAACAGAAATGTCAG
fwd_HEK2-off2	GGAGTGAGTACGGTGTGCGGTACTCGAGTGTTATTCAGCC
rev_HEK2-off2	GAGTTGGATGCTGGATGGGAGCAGTGAGTTTGGGCACT
fwd_HEK3	GGAGTGAGTACGGTGTGCTGCATTTGTAGGCTTGATGC
rev_HEK3	GAGTTGGATGCTGGATGGACATACTAGCCCCTGTCTAG
fwd_HEK3-off1	GGAGTGAGTACGGTGTGCTCCCCTGTTGACCTGGAGAA
rev_HEK3-off1	GAGTTGGATGCTGGATGGTACTTGCCCTGACCAGAGGT
fwd_HEK3-off2	GGAGTGAGTACGGTGTGCGAAGGGGAAGGAGACTTAGTG
rev_HEK3-off2	GAGTTGGATGCTGGATGGTTGGTGTTGACAGGGAGCAA

fwd_HEK3-off3	GGAGTGAGTACGGTGTGCTGCTCATCTTAATCTGCTCAGCC
rev_HEK3-off3	GAGTTGGATGCTGGATGGTCCTAGCACTTTGGAAGGTCG
fwd_HEK3-off4	GGAGTGAGTACGGTGTGCAAAGGAGCAGCTCTTCCTGG
rev_HEK3-off4	GAGTTGGATGCTGGATGGGTCTGCACCATCTCCCACAA
fwd_HEK4	GGAGTGAGTACGGTGTGCAAGATGGCTGACAAAGGCCG
rev_HEK4	GAGTTGGATGCTGGATGGGCCCCCACTGTAGTCACACA
fwd_HEK4-off1	GGAGTGAGTACGGTGTGCGACTCATAGCTGGGGCTGAA
rev_HEK4-off1	GAGTTGGATGCTGGATGGGTATCACTGTCCCCTTGCACT
fwd_HEK4-off2	GGAGTGAGTACGGTGTGCAAGAGGCTGCCCATGAGAGC
rev_HEK4-off2	GAGTTGGATGCTGGATGGTTTGGCAATGGAGGCATTGG
fwd_HEK4-off3	GGAGTGAGTACGGTGTGCTGGGATGGAATCACCTGCAC
rev_HEK4-off3	GAGTTGGATGCTGGATGGCTCGGTTCCTCCACAACACA
fwd_HEK4-off4	GGAGTGAGTACGGTGTGCGCAGGGGAGGGATAAAGCAG
rev_HEK4-off4	GAGTTGGATGCTGGATGGCACGGGAAGGACAGGAGAAG
fwd_Mouse-Tyr	GGAGTGAGTACGGTGTGCCACCGCCCTCTTTTGGAAGT
rev_Mouse-Tyr	GAGTTGGATGCTGGATGGGGCAACTGAGCTTTACCTGAC
fwd_CTNNB1	GGAGTGAGTACGGTGTGCATTTGATGGAGTTGGACATGGCC
rev_ CTNNB1	GAGTTGGATGCTGGATGGCCAGCTACTTGTTCTTGAGTGAAGG
fwd_IP90	GGAGTGAGTACGGTGTGCCTGGTTGACCAATCTGTGGTGAATAG
rev_IP90	GAGTTGGATGCTGGATGGGTACGTACTCAGGCTCATCATCTAACC

Supplementary Table. 12 Primers used for next-generation sequencing.

Primer	Primer sequence (5`-3`)
1F-1	ACTCTTTCCCTACACGACGCTCTTCCGATCTgcttGCGTtggagtgagtacggtgtgc
1F-2	ACTCTTTCCCTACACGACGCTCTTCCGATCTgcttGTAGtggagtgagtacggtgtgc
1F-3	ACTCTTTCCCTACACGACGCTCTTCCGATCTgcttACGCtggagtgagtacggtgtgc
1F-4	ACTCTTTCCCTACACGACGCTCTTCCGATCTgcttCTCGtggagtgagtacggtgtgc
1F-5	ACTCTTTCCCTACACGACGCTCTTCCGATCTgcttGCTCtggagtgagtacggtgtgc
1F-6	ACTCTTTCCCTACACGACGCTCTTCCGATCTgcttAGTCtggagtgagtacggtgtgc
1F-7	ACTCTTTCCCTACACGACGCTCTTCCGATCTgcttCGACtggagtgagtacggtgtgc
1F-8	ACTCTTTCCCTACACGACGCTCTTCCGATCTgcttGATGtggagtgagtacggtgtgc
1F-9	ACTCTTTCCCTACACGACGCTCTTCCGATCTgcttATACtggagtgagtacggtgtgc
1F-10	ACTCTTTCCCTACACGACGCTCTTCCGATCTgcttCACAtggagtgagtacggtgtgc
1F-11	ACTCTTTCCCTACACGACGCTCTTCCGATCTgcttGTGCtggagtgagtacggtgtgc
1F-12	ACTCTTTCCCTACACGACGCTCTTCCGATCTgcttACTAtggagtgagtacggtgtgc
2F	AATGATACGGCGACCACCGAGATCTACACTCTTTCCCTACACGACGCTCTT
1R-A	GACTGGAGTTCAGACGTGTGCTCTTCCGATCTctgtGCGTtgagttggatgctggatgg
1R-B	GACTGGAGTTCAGACGTGTGCTCTTCCGATCTctgtGTAGtgagttggatgctggatgg
1R-C	GACTGGAGTTCAGACGTGTGCTCTTCCGATCTctgtACGCtgagttggatgctggatgg
1R-D	GACTGGAGTTCAGACGTGTGCTCTTCCGATCTctgtCTCGtgagttggatgctggatgg
1R-E	GACTGGAGTTCAGACGTGTGCTCTTCCGATCTctgtGCTCtgagttggatgctggatgg
1R-F	GACTGGAGTTCAGACGTGTGCTCTTCCGATCTctgtAGTCtgagttggatgctggatgg
1R-G	GACTGGAGTTCAGACGTGTGCTCTTCCGATCTctgtCGACtgagttggatgctggatgg
1R-H	GACTGGAGTTCAGACGTGTGCTCTTCCGATCTctgtGATGtgagttggatgctggatgg
2R	CAAGCAGAAGACGGCATACGAGATCGCTGATCGTGACTGGAGTTCAGACGTGTGCTCTT

Supplementary Table. 13 The raw screening data of Library 1.

Mutant	L1-1	L1-2	L1-3	L1-4	L1-5	L1-6	L1-7	L1-8	L1-9	L1-10	L1-11	L1-12	L1-13	L1-14	L1-15	L1-16	L1-17	L1-18	L1-19	L1-20
EGFP(+)%	0.03	2.16	0	0.34	0.62	0.01	2.54	2.28	2.84	3.7	0	0	0	2.15	0.72	1.6	0.14	2.4	0	0
Mutant	L1-21	L1-22	L1-23	L1-24	L1-25	L1-26	L1-27	L1-28	L1-29	L1-30	L1-31	L1-32	L1-33	L1-34	L1-35	L1-36	L1-37	L1-38	L1-39	L1-40
EGFP(+)%	0	2.12	3.68	0	2.47	4.35	0.12	1.6	0	0	0.02	3.6	0.01	0.01	1.47	2.01	2.74	0.12	1.45	0.39
Mutant	L1-41	L1-42	L1-43	L1-44	L1-45	L1-46	L1-47	L1-48	L1-49	L1-50	L1-51	L1-52	L1-53	L1-54	L1-55	L1-56	L1-57	L1-58	L1-59	L1-60
EGFP(+)%	0	9.95	0	3.4	0.62	0.34	0.24	0	0.93	10.18	0	3.46	2.6	1.87	0.14	5.28	5.93	3.24	5.29	12.42
Mutant	L1-61	L1-62	L1-63	L1-64	L1-65	L1-66	L1-67	L1-68	L1-69	L1-70	L1-71	L1-72	L1-73	L1-74	L1-75	L1-76	L1-77	L1-78	L1-79	L1-80
EGFP(+)%	0.48	0	0.11	4.2	4.87	10.97	3.75	5.15	3.89	0.01	4.09	1.08	4.21	0.02	5.29	4.7	4.76	3.22	10.4	0.08
Mutant	L1-81	L1-82	L1-83	L1-84	L1-85	L1-86	L1-87	L1-88	L1-89	L1-90	L1-91	L1-92	L1-93	L1-94	L1-95	L1-96	L1-97	L1-98	L1-99	L1-100
EGFP(+)%	0.03	4.12	0.02	0.02	0.1	5.04	0.03	0	3.34	3.64	0	0	0.64	7.5	0	2.87	0.04	10.84	5.45	0.05
Mutant	L1-101	L1-102	L1-103	L1-104	L1-105	L1-106	L1-107	L1-108	L1-109	L1-110	L1-111	L1-112	L1-113	L1-114	L1-115	L1-116	L1-117	L1-118	L1-119	L1-120
EGFP(+)%	0.01	5.22	4.38	4.28	5.44	0.07	0.02	3.63	0	0	8.25	0.72	0.41	1.48	1.32	0.12	0	1.15	0	2.97
Mutant	L1-121	L1-122	L1-123	L1-124	L1-125	L1-126	L1-127	L1-128	L1-129	L1-130	L1-131	L1-132	L1-133	L1-134	L1-135	L1-136	L1-137	L1-138	L1-139	L1-140
EGFP(+)%	0.86	0.13	0.01	3.47	0.01	1.13	0	4.4	4.42	4.02	0	0.95	2.98	2.1	0	2.79	0.78	0.71	2.68	0
Mutant	L1-141	L1-142	L1-143	L1-144	L1-145	L1-146	L1-147	L1-148	L1-149	L1-150	L1-151	L1-152	L1-153	L1-154	L1-155	L1-156	L1-157	L1-158	L1-159	L1-160
EGFP(+)%	0.1	0	2.86	0.02	0.18	0.08	0	2.17	3.41	0.96	0.05	0.01	0.01	3.63	0.01	0.01	0	0	3.24	0.27
Mutant	L1-161	L1-162	L1-163	L1-164	L1-165	L1-166	L1-167	L1-168	L1-169	L1-170	L1-171	L1-172	L1-173	L1-174	L1-175	L1-176	L1-177	L1-178	L1-179	L1-180
EGFP(+)%	1.42	1.66	2.57	0.03	4.43	0.04	0.56	2.75	1.62	0.08	0.01	2.65	3.19	2.36	2.96	3.05	3.93	4.05	1.19	3.47
Mutant	L1-181	L1-182	L1-183	L1-184	L1-185	L1-186	L1-187	L1-188	L1-189	L1-190	L1-191	L1-192	L1-193	L1-194	L1-195	L1-196	L1-197	L1-198	L1-199	L1-200
EGFP(+)%	0.22	0.77	1.8	0	1.94	0.89	0.34	1.94	0.26	0.4	2.56	2.77	0.15	2.86	2.56	2.45	2.69	0.01	3.3	0.72
Mutant	L1-201	L1-202	L1-203	L1-204	L1-205	L1-206	L1-207	L1-208	L1-209	L1-210	L1-211	L1-212	L1-213	L1-214	L1-215	L1-216	L1-217	L1-218	L1-219	L1-220
EGFP(+)%	2.81	0.01	0.85	0.02	0.02	0.01	0.01	3.11	1.9	2.87	0.04	0.11	0.01	2.97	1.13	0.13	2.64	0.11	3.26	3.41
Mutant	L1-221	L1-222	L1-223	L1-224	L1-225	L1-226	L1-227	L1-228	L1-229	L1-230	L1-231	L1-232	L1-233	L1-234	L1-235	L1-236	L1-237	L1-238	L1-239	L1-240
EGFP(+)%	3.34	0.01	3.53	3.37	1.13	2.4	3.58	2.42	3.67	2.13	3.37	0.01	4.04	0.65	4	0.64	0.05	0.01	3.3	11.46
Mutant	L1-241	L1-242	L1-243	L1-244	L1-245	L1-246	L1-247	L1-248	L1-249	L1-250	L1-251	L1-252	L1-253	L1-254	L1-255	L1-256	L1-257	L1-258	L1-259	L1-260
EGFP(+)%	0	0.17	0	11.41	0.01	4.35	0	0.68	4.24	0	0.03	3.96	2.77	0.76	0.79	8.08	3.39	2.41	2.3	0
Mutant	L1-261	L1-262	L1-263	L1-264	L1-265	L1-266	L1-267	L1-268	L1-269	L1-270	L1-271	L1-272	L1-273	L1-274	L1-275	L1-276	L1-277	L1-278	L1-279	L1-280
EGFP(+)%	2.79	0	4.08	3.75	3.34	2.78	0.01	4.35	1.36	0.28	4.32	5.68	5.19	3.21	1.48	0	0.04	1.68	0.07	0
Mutant	L1-281	L1-282	L1-283	L1-284	L1-285	L1-286	L1-287	L1-288	L1-289	L1-290	L1-291	L1-292	L1-293	L1-294	L1-295	L1-296	L1-297	L1-298	L1-299	L1-300
EGFP(+)%	2.07	0.03	0.45	0.45	4.76	0	2.19	2.06	2.45	2.46	0.02	0.06	3.76	2.22	4.65	3.49	3.94	0	0.01	2.79
Mutant	L1-301	L1-302	L1-303	L1-304	L1-305	L1-306	L1-307	L1-308	L1-309	L1-310	L1-311	L1-312	L1-313	L1-314	L1-315	L1-316	L1-317	L1-318	L1-319	L1-320
EGFP(+)%	1.21	1.21	0	0.71	0	0.03	0	0.02	0.01	2.14	0	0.01	2.25	3.91	0.01	3.61	0.52	3.29	0.26	0.03
Mutant	L1-321	L1-322	L1-323	L1-324	L1-325	L1-326	L1-327	L1-328	L1-329	L1-330	L1-331	L1-332	L1-333	L1-334	L1-335	L1-336	L1-337	L1-338	L1-339	L1-340
EGFP(+)%	3.22	0.02	3.93	4.13	0.01	1.61	2	0.15	1.15	2.5	1.88	0.61	2.35	0.02	3.67	0.25	0.03	0.13	0.37	2.95
Mutant	L1-341	L1-342	L1-343	L1-344	L1-345	L1-346	L1-347	L1-348	L1-349	L1-350	L1-351	L1-352	L1-353	L1-354	L1-355	L1-356	L1-357	L1-358	L1-359	L1-360
EGFP(+)%	0.01	0	1.02	3.75	0.02	0.17	4.17	0.63	0.01	2.81	0.05	0.02	0.03	0.04	1.3	0.32	2.83	0.01	6.32	3.16
Mutant	L1-361	L1-362	L1-363	L1-364	L1-365	L1-366	L1-367	L1-368	L1-369	L1-370	L1-371	L1-372	L1-373	L1-374	L1-375	L1-376	L1-377	L1-378	L1-379	L1-380
EGFP(+)%	0	3	0.52	0.07	0	0.19	2.91	1.18	0.53	2.26	2.34	3.3	2.75	1.98	2.39	2.76	2.17	2.59	2.01	0.02
Mutant	L1-381	L1-382	L1-383	L1-384	L1-385	L1-386	L1-387	L1-388	L1-389	L1-390	L1-391	L1-392	L1-393	L1-394	L1-395	L1-396	L1-397	L1-398	L1-399	L1-400
EGFP(+)%	0.02	1.65	0.66	2.52	0	7.88	0.69	0.02	2.58	0.5	2.48	4.48	6.11	0.06	3.02	0	0	2.38	1.42	1.09
Mutant	L1-401	L1-402	L1-403	L1-404	L1-405	L1-406	L1-407	L1-408	L1-409	L1-410	L1-411	L1-412	L1-413	L1-414	L1-415	L1-416	L1-417	L1-418	L1-419	L1-420
EGFP(+)%	1.56	2.3	0.11	0.4	1.19	2.57	1.02	2.29	0.03	5.73	2.17	2	0.01	0	2.27	0	1.08	1.31	0.31	0.02
Mutant	L1-421	L1-422	L1-423	L1-424	L1-425	L1-426	L1-427	L1-428	L1-429	L1-430	L1-431	L1-432	L1-433	L1-434	L1-435	L1-436	L1-437	L1-438	L1-439	L1-440
EGFP(+)%	0	0.89	1.85	1.61	0.07	2.76	1.61	0	0	0.32	2.62	0	0.01	0.68	2.4	2.64	5.02	1.81	0.04	2.78
Mutant	L1-441	L1-442	L1-443	L1-444	L1-445	L1-446	L1-447	L1-448	L1-449	L1-450	L1-451	L1-452	L1-453	L1-454	L1-455	L1-456	L1-457	L1-458	L1-459	L1-460
EGFP(+)%	1.91	2.39	0	0.01	0.05	2.69	3.44	0	0	0	0	0.07	2.78	0	4.06	0	0.1	1.83	1.65	0.02
Mutant	L1-461	L1-462	L1-463	L1-464	L1-465	L1-466	L1-467	L1-468	L1-469	L1-470	L1-471	L1-472	L1-473	L1-474	L1-475	L1-476	L1-477	L1-478	L1-479	L1-480
EGFP(+)%	0.08	1.24	4.26	5.36	2.89	1.57	3.52	0.02	2.08	0.08	2.64	0.06	0.17	0.04	0	0.05	0.6	3.52	0.36	2.9
Mutant	L1-481	L1-482	L1-483	L1-484	L1-485	L1-486	L1-487	L1-488	L1-489	L1-490	L1-491	L1-492	L1-493	L1-494	L1-495	L1-496	L1-497	L1-498	L1-499	L1-500
EGFP(+)%	0.1	0	0.03	4.19	0.12	0.03	0.06	0	4.66	0	0	0.12	0.16	2.68	0	0	0.39	0.04	6.58	0.05
Mutant	L1-501	L1-502	L1-503	L1-504	L1-505	L1-506	L1-507	L1-508	L1-509	L1-510	L1-511	L1-512	L1-513	L1-514	L1-515	L1-516	L1-517	L1-518	L1-519	L1-520
EGFP(+)%	0.18	3.96	4.89	0.26	0	0.01	0	0	0	0	0	1.86	0.7	0.03	0.04	0.02	0.02	2.12	0	4.48
Mutant	L1-521	L1-522	L1-523	L1-524	L1-525	L1-526	L1-527	L1-528	L1-529	L1-530	L1-531	L1-532	L1-533	L1-534	L1-535	L1-536	L1-537	L1-538	L1-539	L1-540
EGFP(+)%	2.97	0.48	0	2.93	0.18	3.53	0.08	0	0.35	0.45	2.86	3.37	5.07	4.17	1.06	2.34	5.14	0.02	0.01	0.07
Mutant	L1-541	L1-542	L1-543	L1-544	L1-545	L1-546	L1-547	L1-548	L1-549	L1-550	L1-551	L1-552	L1-553	L1-554	L1-555	L1-556	L1-557	L1-558	L1-559	L1-560
EGFP(+)%	3.52	0	1.24	0	0	0.99	0.13	0.11	0.01	2.45	0.05	0.03	1.51	0.01	0.69	2.69	0.27	4.09	2.49	0.61
Mutant	L1-561	L1-562	L1-563	L1-564	L1-565	L1-566	L1-567	L1-568	L1-569	L1-570	L1-571	L1-572	L1-573	L1-574	L1-575	L1-576	L1-577	L1-578	L1-579	L1-580
EGFP(+)%	0	1.32	0	0.33	0.37	0.15	0.01	0	1.37	2.42	0.01	0.01	4.94	4.92	7.25	0.11	3.21	5.34	0.05	0.14
Mutant	L1-581	L1-582	L1-583	L1-584	L1-585	L1-586	L1-587	L1-588	L1-589	L1-590	L1-591	L1-592	L1-593	L1-594	L1-595	L1-596	L1-597	L1-598	L1-599	L1-600
EGFP(+)%	3.41	2.23	4.28	0.01	0.03	4.57	0.03	5.43	3.34	5.58	0.02	0.02	0	1.01	0.07	0.01	2.97	0.59	1.03	0.01
Mutant	L1-601	L1-602	L1-603	L1-604	L1-605	L1-606	L1-607	L1-608	L1-609	L1-610	L1-611	L1-612	L1-613	L1-614	L1-615	L1-616	L1-617	L1-618	L1-619	L1-620
EGFP(+)%	2.21	2.16	0.54	3.79	3.13	0.01	2.52	0.15	0.64	0.21	0.03	0.02	0	0.01	0	3.08	4.69	3.23	4.93	4.51
Mutant	L1-621	L1-622	L1-623	L1-624	L1-625	L1-626	L1-627	L1-628	L1-629	L1-630	L1-631	L1-632	L1-633	L1-634	L1-635	L1-636	L1-637	L1-638	L1-639	L1-640
EGFP(+)%	0.07	4.11	0.49	0.01	5.13	5.93	0.22	2.87	0.03	0.23	1.46	0.05	0	2.52	5.94	6.36	3.07	0.07	2.08	2.4
Mutant	L1-641	L1-642	L1-643	L1-644	L1-645	L1-646	L1-647	L1-648	L1-649	L1-650	L1-651	L1-652	L1-653	L1-654	L1-655	L1-656	L1-657	L1-658	L1-659	L1-660
EGFP(+)%	1.02	2.04	0.01	0.04	0.08	0.01	0.13	0.14	0.01	0.06	0.73	0.17	3.01	3.19	2.91	0	1.41	2.11	0	0
Mutant	L1-661	L1-662	L1-663	L1-664	L1-665	L1-666	L1-667	L1-668	L1-669	L1-670	L1-671	L1-672	L1-673	L1-674	L1-675	L1-676	L1-677	L1-678	L1-679	L1-680
EGFP(+)%	1.88	0.08	2.54	0	0.02	3.08	0	0.08	1.69	2.04	0.25	0.01	0	0.01	0.01	1.83	1.69	3.7	6.37	2.22
Mutant	L1-681	L1-682	L1-683	L1-684	L1-685	L1-686	L1-687	L1-688	L1-689	L1-690	L1-691	L1-692	L1-693	L1-694	L1-695	L1-696	L1-697	L1-698	L1-699	L1-700
EGFP(+)%	1.05	0	0.48	0.01	2.59	0.4	4.08	0	1.78	0.38	0	0.04	4.46	0.03	0.01	3.96	1.59	1.97	0.08	0
Mutant	L1-701	L1-702	L1-703	L1-704	L1-705	L1-706	L1-707	L1-708	L1-709	L1-710	L1-711	L1-712	L1-713	L1-714	L1-715	L1-716	L1-717	L1-718	L1-719	L1-720
EGFP(+)%	0.37	0.1	0.94	5.28	0.2	2.39	4.26	5.85	0.01	5.49	0.08	0.02	2.19	6.05	1.38	0.01	0.71	6.18	0.04	0
Mutant	L1-721	L1-722	L1-723	L1-724	L1-725	L1-726	L1-727	L1-728	L1-729	L1-730	L1-731	L1-732	L1-733	L1-734	L1-735	L1-736	L1-737	L1-738	L1-739	L1-740
EGFP(+)%	2.51	3.7	0.16	1.81	0.51	0.88	2.6	0.08	0	3.81	0.04	6.11	0.02	1.52	3.26	0.06	0	1.01	1.06	1.54
Mutant	L1-741	L1-742	L1-743	L1-744	L1-745	L1-746	L1-747	L1-748	L1-749	L1-750	L1-751	L1-752	L1-753	L1-754	L1-755	L1-756	L1-757	L1-758	L1-759	L1-760
EGFP(+)%	4.09	0.11	0.02	1.73	2.62	4.2	3.2	1.35	4.26	2.82	8.92	4.19	0.19	4.19	0.1	0.11	6.3	1.02	0.12	5.23
Mutant	L1-761	L1-762	L1-763	L1-764	L1-765	L1-766	L1-767	L1-768	L1-769	L1-770	L1-771	L1-772	L1-773	L1-774	L1-775	L1-776	L1-777	L1-778	L1-779	L1-780
EGFP(+)%	7.08	6.01	5.41	0.21	5.65	4.14	2.2	1.2	0.27	0	2.8	0.01	2.32	0.07	1.37	2.78	0.57	0	0.01	0

Mutant	L1-781	L1-782	L1-783	L1-784	L1-785	L1-786	L1-787	L1-788	L1-789	L1-790	L1-791	L1-792	L1-793	L1-794	L1-795	L1-796	L1-797	L1-798	L1-799	L1-800
EGFP(+)%	1.53	3.84	1.25	2.56	0.02	0.6	2	3.82	4.05	0.88	0.05	2.14	3.23	0.05	0	3.46	0.05	3.45	0.02	3.52
Mutant	L1-801	L1-802	L1-803	L1-804	L1-805	L1-806	L1-807	L1-808	L1-809	L1-810	L1-811	L1-812	L1-813	L1-814	L1-815	L1-816	L1-817	L1-818	L1-819	L1-820
EGFP(+)%	1.42	3.86	1.99	1.72	0.04	3.89	0	0.08	1.63	0.1	7.28	3.39	0.33	1.74	5.12	1.39	2.56	0.08	0.01	3.4
Mutant	L1-821	L1-822	L1-823	L1-824	L1-825	L1-826	L1-827	L1-828	L1-829	L1-830	L1-831	L1-832	L1-833	L1-834	L1-835	L1-836	L1-837	L1-838	L1-839	L1-840
EGFP(+)%	3.88	0.02	0.82	0.58	0.02	0	2.26	2.63	3.78	0.03	0.01	3.37	0.74	0.04	3.6	0	3.14	3.17	0.04	5.08
Mutant	L1-841	L1-842	L1-843	L1-844	L1-845	L1-846	L1-847	L1-848	L1-849	L1-850	L1-851	L1-852	L1-853	L1-854	L1-855	L1-856	L1-857	L1-858	L1-859	L1-860
EGFP(+)%	0	4.55	0.81	0.28	1.9	4.27	2.61	0.56	3.52	3.69	3.89	0	2.59	0	0.33	2.11	0	1.26	2.15	0
Mutant	L1-861	L1-862	L1-863	L1-864	L1-865	L1-866	L1-867	L1-868	L1-869	L1-870	L1-871	L1-872	L1-873	L1-874	L1-875	L1-876	L1-877	L1-878	L1-879	L1-880
EGFP(+)%	1.27	0.02	0.22	3.02	3.46	0	0	3.3	3.43	3.34	0.04	1.92	1.36	4.12	2.38	0.13	0	3.54	0	0.04
Mutant	L1-881	L1-882	L1-883	L1-884	L1-885	L1-886	L1-887	L1-888	L1-889	L1-890	L1-891	L1-892	L1-893	L1-894	L1-895	L1-896	L1-897	L1-898	L1-899	L1-900
EGFP(+)%	0	3.36	1.9	0.53	2.63	1.02	0.03	1.62	1.91	6.87	3.15	1.15	2.74	0.13	0	3.73	0.02	0.03	0.15	0.66
Mutant	L1-901	L1-902	L1-903	L1-904	L1-905	L1-906	L1-907	L1-908	L1-909	L1-910	L1-911	L1-912	L1-913	L1-914	L1-915	L1-916	L1-917	L1-918	L1-919	L1-920
EGFP(+)%	0.9	0	0.02	0.01	1.26	2.91	1.15	4.09	0.02	3.12	3.11	2.94	1.38	0.14	1.15	0.01	1.85	2.65	0.11	2.35
Mutant	L1-921	L1-922	L1-923	L1-924	L1-925	L1-926	L1-927	L1-928	L1-929	L1-930	L1-931	L1-932	L1-933	L1-934	L1-935	L1-936	L1-937	L1-938	L1-939	L1-940
EGFP(+)%	0.71	0.05	0.14	1.3	2.45	0.01	2.67	0.25	1.51	2.22	1.15	0.84	1.13	1.47	0.05	1.08	0.17	0	0	0.01
Mutant	L1-941	L1-942	L1-943	L1-944	L1-945	L1-946	L1-947	L1-948	L1-949	L1-950	L1-951	L1-952	L1-953	L1-954	L1-955	L1-956	L1-957	L1-958	L1-959	L1-960
EGFP(+)%	0	0.01	2.39	0.12	1.53	3.91	0.51	0.29	0.39	1.96	0.39	0.01	0	1.05	2.72	3.79	1.67	0	0	0
Mutant	L1-961	L1-962	L1-963	L1-964	L1-965	L1-966	L1-967	L1-968	L1-969	L1-970	L1-971	L1-972	L1-973	L1-974	L1-975	L1-976	L1-977	L1-978	L1-979	L1-980
EGFP(+)%	3.31	0	0.04	0.02	0.24	3.85	0.05	4.32	0.11	0.01	1.61	0.69	4.2	4.36	4.39	4.12	3.83	0.05	0.13	1.47
Mutant	L1-981	L1-982	L1-983	L1-984	L1-985	L1-986	L1-987	L1-988	L1-989	L1-990	L1-991	L1-992	L1-993	L1-994	L1-995	L1-996	L1-997	L1-998	L1-999	L1-1000
EGFP(+)%	4.87	0.01	0	0.99	5.84	0.01	0	0.02	0	0.03	2.06	0.07	4.66	3.61	0.15	0.7	3.58	0.13	3.97	0.21
Mutant	L1-1001	L1-1002	L1-1003	L1-1004	L1-1005	L1-1006	L1-1007	L1-1008	L1-1009	L1-1010	L1-1011	L1-1012	L1-1013	L1-1014	L1-1015	L1-1016	L1-1017	L1-1018	L1-1019	L1-1020
EGFP(+)%	0	4.16	0	1.75	1.91	0.57	0	1.79	0.13	0	0.8	0.02	0.04	0.12	1.84	1.74	0	2.16	0.62	2.21
Mutant	L1-1021	L1-1022	L1-1023	L1-1024	L1-1025	L1-1026	L1-1027	L1-1028	L1-1029	L1-1030	L1-1031	L1-1032	L1-1033	L1-1034	L1-1035	L1-1036	L1-1037	L1-1038	L1-1039	L1-1040
EGFP(+)%	0.64	0	0.02	0	0	6.3	5.36	0.35	0.6	0.02	7.67	8.22	2.66	3.3	4.54	0.14	2.28	1.58	2.11	0.48
Mutant	L1-1041	L1-1042	L1-1043	L1-1044	L1-1045	L1-1046	L1-1047	L1-1048	L1-1049	L1-1050	L1-1051	L1-1052	L1-1053	L1-1054	L1-1055	L1-1056	L1-1057	L1-1058	L1-1059	L1-1060
EGFP(+)%	0.3	0.71	0.01	0.37	0	0.22	0	1.27	0	0	3.89	4.38	0.6	0.05	0	1.41	1.1	0	2.92	0
Mutant	L1-1061	L1-1062	L1-1063	L1-1064	L1-1065	L1-1066	L1-1067	L1-1068	L1-1069	L1-1070	L1-1071	L1-1072	L1-1073	L1-1074	L1-1075	L1-1076	L1-1077	L1-1078	L1-1079	L1-1080
EGFP(+)%	0.07	2.8	0	6.55	1.59	0.11	0.19	3	1.1	1.8	0	0.05	0.07	2.99	0.03	0	2.79	0.45	1.91	2.92
Mutant	L1-1081	L1-1082	L1-1083	L1-1084	L1-1085	L1-1086	L1-1087	L1-1088	L1-1089	L1-1090	L1-1091	L1-1092	L1-1093	L1-1094	L1-1095	L1-1096	L1-1097	L1-1098	L1-1099	L1-1100
EGFP(+)%	2.64	3.98	2.97	4.4	1.68	2.2	0	1.9	5.02	0.84	0.3	4.33	0	0	0	0	3.98	0.06	0.23	1.89
Mutant	L1-1101	L1-1102	L1-1103	L1-1104	L1-1105	L1-1106	L1-1107	L1-1108	L1-1109	L1-1110	L1-1111	L1-1112	L1-1113	L1-1114	L1-1115	L1-1116	L1-1117	L1-1118	L1-1119	L1-1120
EGFP(+)%	1.5	0	0	0	1.72	0	1.95	1.3	0.91	1.71	2.81	2.13	3.93	0	0	3.67	0	0	0	0
Mutant	L1-1121	L1-1122	L1-1123	L1-1124	L1-1125	L1-1126	L1-1127	L1-1128	L1-1129	L1-1130	L1-1131	L1-1132	L1-1133	L1-1134	L1-1135	L1-1136	L1-1137	L1-1138	L1-1139	L1-1140
EGFP(+)%	0	0	0	0	2.59	0	0.07	0.29	0.24	0.08	1119	0.36	0.04	0.05	0.04	0.19	6.77	0.16	5.55	4.81
Mutant	L1-1141	L1-1142	L1-1143	L1-1144	L1-1145	L1-1146	L1-1147	L1-1148	L1-1149	L1-1150	L1-1151	L1-1152	L1-1153	L1-1154	L1-1155	L1-1156	L1-1157	L1-1158	L1-1159	L1-1160
EGFP(+)%	0	0.31	0.39	6.64	0.17	3.08	5.71	3.01	1.43	5.77	1.61	2.15	2.59	1.2	0.26	1.52	0.1	0	2.36	0.62
Mutant	L1-1161	L1-1162	L1-1163	L1-1164	L1-1165	L1-1166	L1-1167	L1-1168	L1-1169	L1-1170	L1-1171	L1-1172	L1-1173	L1-1174	L1-1175	L1-1176	L1-1177	L1-1178	L1-1179	L1-1180
EGFP(+)%	2.49	4.08	0.08	0.01	5.19	0	0.03	4.4	0.52	0	0.03	4.47	0.37	0	0.58	3.31	0.08	0	0.05	0.63
Mutant	L1-1181	L1-1182	L1-1183	L1-1184	L1-1185	L1-1186	L1-1187	L1-1188	L1-1189	L1-1190	L1-1191	L1-1192	L1-1193	L1-1194	L1-1195	L1-1196	L1-1197	L1-1198	L1-1199	L1-1200
EGFP(+)%	0.08	5.88	7.94	0.04	2.24	4.29	1.98	3.91	5.72	2.46	0.37	0.37	0.18	1.31	0.05	3.86	0.01	2.24	0.37	5.52
Mutant	L1-1201	L1-1202	L1-1203	L1-1204	L1-1205	L1-1206	L1-1207	L1-1208	L1-1209	L1-1210	L1-1211	L1-1212	L1-1213	L1-1214	L1-1215	L1-1216	L1-1217	L1-1218	L1-1219	L1-1220
EGFP(+)%	0.03	3.11	3.18	4.27	0.49	1.24	4.01	0	4.08	1.95	3.83	0.09	5.34	0.02	0.05	0.21	3.85	2.75	0.44	0.02
Mutant	L1-1221	L1-1222	L1-1223	L1-1224	L1-1225	L1-1226	L1-1227	L1-1228	L1-1229	L1-1230	L1-1231	L1-1232	L1-1233	L1-1234	L1-1235	L1-1236	L1-1237	L1-1238	L1-1239	L1-1240
EGFP(+)%	2.15	1.48	2.91	0	0	0.09	0	0.06	2.79	0	0	2.92	0	1.41	0	0.02	0.41	0.02	0.88	0.09
Mutant	L1-1241	L1-1242	L1-1243	L1-1244	L1-1245	L1-1246	L1-1247	L1-1248	L1-1249	L1-1250	L1-1251	L1-1252	L1-1253	L1-1254	L1-1255	L1-1256	L1-1257	L1-1258	L1-1259	L1-1260
EGFP(+)%	2.54	0.43	3.93	0.01	2.17	1.46	0.34	0.69	0.04	0	0.06	0.03	4.79	5.31	0.42	0.96	0.01	0.29	6.77	0
Mutant	L1-1261	L1-1262	L1-1263	L1-1264	L1-1265	L1-1266	L1-1267	L1-1268	L1-1269	L1-1270	L1-1271	L1-1272	L1-1273	L1-1274	L1-1275	L1-1276	L1-1277	L1-1278	L1-1279	L1-1280
EGFP(+)%	0.03	4.75	4.72	0.5	0.06	0.04	2.57	0.12	0.01	0.99	1.4	0.99	0.11	0.05	6.66	1.08	0	5.74	5.35	0.15
Mutant	L1-1281	L1-1282	L1-1283	L1-1284	L1-1285	L1-1286	L1-1287	L1-1288	L1-1289	L1-1290	L1-1291	L1-1292	L1-1293	L1-1294	L1-1295	L1-1296	L1-1297	L1-1298	L1-1299	L1-1300
EGFP(+)%	1.92	2.57	0.32	3.92	0.58	3.74	0	0.02	0.1	3.04	0.02	2.82	0.24	0	5.32	3.86	0.38	0.55	0	3.08
Mutant	L1-1301	L1-1302	L1-1303	L1-1304	L1-1305	L1-1306	L1-1307	L1-1308	L1-1309	L1-1310	L1-1311	L1-1312	L1-1313	L1-1314	L1-1315	L1-1316	L1-1317	L1-1318	L1-1319	L1-1320
EGFP(+)%	0.36	1.86	0	2.29	0	0.5	0	0	0	0.11	0.77	0.03	1.79	0	0.04	0	0	0.37	2.07	0.89
Mutant	L1-1321	L1-1322	L1-1323	L1-1324	L1-1325	L1-1326	L1-1327	L1-1328	L1-1329	L1-1330	L1-1331	L1-1332	L1-1333	L1-1334	L1-1335	L1-1336	L1-1337	L1-1338	L1-1339	L1-1340
EGFP(+)%	1.04	0.44	0	0.14	0	1.71	0	3.26	1.79	1	1.34	0	0	0	4.74	3.65	2.61	0.5	1.13	0.33
Mutant	L1-1341	L1-1342	L1-1343	L1-1344	L1-1345	L1-1346	L1-1347	L1-1348	L1-1349	L1-1350	L1-1351	L1-1352	L1-1353	L1-1354	L1-1355	L1-1356	L1-1357	L1-1358	L1-1359	L1-1360
EGFP(+)%	0.02	0	4.32	2.37	0	0.59	0.06	2.04	0.8	0	4.34	2.19	7.57	4.24	3.51	0	0.18	2.71	2.06	0.36
Mutant	L1-1361	L1-1362	L1-1363	L1-1364	L1-1365	L1-1366	L1-1367	L1-1368	L1-1369	L1-1370	L1-1371	L1-1372	L1-1373	L1-1374	L1-1375	L1-1376	L1-1377	L1-1378	L1-1379	L1-1380
EGFP(+)%	2.43	1.16	2.97	0	2.25	0	0.33	0	0	3.44	3.49	0	3.65	2.07	0.25	0	2.96	2.95	0.2	0
Mutant	L1-1381	L1-1382	L1-1383	L1-1384	L1-1385	L1-1386	L1-1387	L1-1388	L1-1389	L1-1390	L1-1391	L1-1392	L1-1393	L1-1394	L1-1395	L1-1396	L1-1397	L1-1398	L1-1399	L1-1400
EGFP(+)%	1.02	1.15	0.03	0.02	0.45	0.1	1.37	0.8	0	0.01	0.41	3.99	2.96	0	0	0.2	0	1.45	1.74	1.1
Mutant	L1-1401	L1-1402	L1-1403	L1-1404	L1-1405	L1-1406	L1-1407	L1-1408	L1-1409	L1-1410	L1-1411	L1-1412	L1-1413	L1-1414	L1-1415	L1-1416	L1-1417	L1-1418	L1-1419	L1-1420
EGFP(+)%	3.04	0	0.8	0	0.08	1.25	1.7	0.22	0	0.92	0	0	1.36	0.02	2.44	0	2.21	1.24	1.08	0.02
Mutant	L1-1421	L1-1422	L1-1423	L1-1424	L1-1425	L1-1426	L1-1427	L1-1428												
EGFP(+)%	0	4.37	3.39	0.54	2.58	3.31	2.08	1.65												

Supplementary Table. 14 The raw screening data of Library 2.

Mutant	L2-1	L2-2	L2-3	L2-4	L2-5	L2-6	L2-7	L2-8	L2-9	L2-10	L2-11	L2-12	L2-13	L2-14	L2-15	L2-16	L2-17	L2-18	L2-19	L2-20
EGFP (+)%	0.48	1.38	2.73	2.73	2.84	5.86	0.46	1.7	5.12	3.59	2.14	4.45	1.64	3.91	3.87	0.54	4.01	3.7	2.4	4.14
Mutant	L2-21	L2-22	L2-23	L2-24	L2-25	L2-26	L2-27	L2-28	L2-29	L2-30	L2-31	L2-32	L2-33	L2-34	L2-35	L2-36	L2-37	L2-38	L2-39	L2-40
EGFP (+)%	3.22	2.86	1.3	4.39	2.68	4.19	0.06	0.94	2.01	3.35	2.37	2.59	2.96	2.81	8.26	2.31	2.36	0.03	2.91	4.32
Mutant	L2-41	L2-42	L2-43	L2-44	L2-45	L2-46	L2-47	L2-48	L2-49	L2-50	L2-51	L2-52	L2-53	L2-54	L2-55	L2-56	L2-57	L2-58	L2-59	L2-60
EGFP (+)%	1.64	4.06	0.32	1.26	1.94	0.02	2.42	0	2.93	2.37	2.35	1.25	2.17	1.37	2.8	1.14	1.35	2.13	5	0.09
Mutant	L2-61	L2-62	L2-63	L2-64	L2-65	L2-66	L2-67	L2-68	L2-69	L2-70	L2-71	L2-72	L2-73	L2-74	L2-75	L2-76	L2-77	L2-78	L2-79	L2-80
EGFP (+)%	3.57	2.7	0.83	0.67	0.56	1.65	0.76	3.91	2.81	2.56	0.15	1.71	0.89	2.49	2.7	2.26	2.41	2.56	2.6	0.69
Mutant	L2-81	L2-82	L2-83	L2-84	L2-85	L2-86	L2-87	L2-88	L2-89	L2-90	L2-91	L2-92	L2-93	L2-94	L2-95	L2-96	L2-97	L2-98	L2-99	L2-100
EGFP (+)%	4.23	4.31	3.57	1.4	2.26	0.59	2.42	3.08	0	3.42	3.34	3.85	2.68	1.32	2.77	2.72	0.04	0.56	1.97	1.81
Mutant	L2-101	L2-102	L2-103	L2-104	L2-105	L2-106	L2-107	L2-108	L2-109	L2-110	L2-111	L2-112	L2-113	L2-114	L2-115	L2-116	L2-117	L2-118	L2-119	L2-120
EGFP (+)%	3.44	0.39	7.89	1.75	3.92	9.06	3.03	2.6	4.83	0.12	2.29	2.61	4.8	4.73	2.85	3.08	1.6	8.9	0.13	0.09
Mutant	L2-121	L2-122	L2-123	L2-124	L2-125	L2-126	L2-127	L2-128	L2-129	L2-130	L2-131	L2-132	L2-133	L2-134	L2-135	L2-136	L2-137	L2-138	L2-139	L2-140
EGFP (+)%	0.45	2.91	0.14	5.37	3.67	3.43	1.5	3.53	1.11	3.56	5.7	1.77	3.81	4.99	6.1	2.63	3.37	2.38	1.42	6.17
Mutant	L2-141	L2-142	L2-143	L2-144	L2-145	L2-146	L2-147	L2-148	L2-149	L2-150	L2-151	L2-152	L2-153	L2-154	L2-155	L2-156	L2-157	L2-158	L2-159	L2-160
EGFP (+)%	3.27	6.26	3.26	1.23	5.1	3.63	0.25	4.27	1.6	4.72	10.71	0.15	4.1	2.1	1.82	11.29	2.84	3.89	10.21	1.79
Mutant	L2-161	L2-162	L2-163	L2-164	L2-165	L2-166	L2-167	L2-168	L2-169	L2-170	L2-171	L2-172	L2-173	L2-174	L2-175	L2-176	L2-177	L2-178	L2-179	L2-180
EGFP (+)%	3.27	7.85	8.6	4.8	1.77	5.05	1.9	8.25	3.4	5.52	8.31	1.52	1.42	2.94	1.03	1.74	3.03	6.59	1.84	0.01
Mutant	L2-181	L2-182	L2-183	L2-184	L2-185	L2-186	L2-187	L2-188	L2-189	L2-190	L2-191	L2-192	L2-193	L2-194	L2-195	L2-196	L2-197	L2-198	L2-199	L2-200
EGFP (+)%	2.95	1.54	2.35	2.13	3.24	2.3	1.6	2.36	2.48	2.5	1.74	1.69	2.17	1.47	2.04	2.15	2.87	1	1.62	2.35
Mutant	L2-201	L2-202	L2-203	L2-204	L2-205	L2-206	L2-207	L2-208	L2-209	L2-210	L2-211	L2-212	L2-213	L2-214	L2-215	L2-216	L2-217	L2-218	L2-219	L2-220
EGFP (+)%	4.02	1.98	0	1.85	3.56	3.58	3.98	1.96	0.75	3.87	2.62	2.57	1.23	2.43	1.87	0.83	1.65	1.15	4.35	1.19
Mutant	L2-221	L2-222	L2-223	L2-224	L2-225	L2-226	L2-227	L2-228	L2-229	L2-230	L2-231	L2-232	L2-233	L2-234	L2-235	L2-236	L2-237	L2-238	L2-239	L2-240
EGFP (+)%	0.98	1.6	3.46	2.2	2.18	2.68	0.49	3.26	1.2	0.21	2.24	0.87	0.34	2	0	3.08	1.07	3.41	2.53	3.27
Mutant	L2-241	L2-242	L2-243	L2-244	L2-245	L2-246	L2-247	L2-248	L2-249	L2-250	L2-251	L2-252	L2-253	L2-254	L2-255	L2-256	L2-257	L2-258	L2-259	L2-260
EGFP (+)%	0	2.54	0.09	2.31	3.6	1.23	3.51	1.32	1.35	3.18	2.34	1.74	3.92	2.91	2.17	0.31	3.32	3.14	0.06	3.26
Mutant	L2-261	L2-262	L2-263	L2-264	L2-265	L2-266	L2-267	L2-268	L2-269	L2-270	L2-271	L2-272	L2-273	L2-274	L2-275	L2-276	L2-277	L2-278	L2-279	L2-280
EGFP (+)%	3.1	2.58	0.26	6.31	2.44	0.32	1.07	2.11	0.69	2.06	1.42	0.13	2.46	1.67	5.51	1.85	2.05	1.56	3.2	1.87
Mutant	L2-281	L2-282	L2-283	L2-284	L2-285	L2-286	L2-287	L2-288	L2-289	L2-290	L2-291	L2-292	L2-293	L2-294	L2-295	L2-296	L2-297	L2-298	L2-299	L2-300
EGFP (+)%	2.1	1.97	0.5	2.56	0.35	2.22	2.98	3.24	4.28	0	1.43	1.73	1.98	0.6	3.28	0.03	3.11	0.22	2.35	4.67
Mutant	L2-301	L2-302	L2-303	L2-304	L2-305	L2-306	L2-307	L2-308	L2-309	L2-310	L2-311	L2-312	L2-313	L2-314	L2-315	L2-316	L2-317	L2-318	L2-319	L2-320
EGFP (+)%	1.58	1.08	3.72	2.41	0.11	1.42	1.58	2.22	0.29	0.27	2.17	0.4	4.75	1.79	0.02	2.25	0.05	2.48	3.15	8.59
Mutant	L2-321	L2-322	L2-323	L2-324	L2-325	L2-326	L2-327	L2-328	L2-329	L2-330	L2-331	L2-332	L2-333	L2-334	L2-335	L2-336	L2-337	L2-338	L2-339	L2-340
EGFP (+)%	0	2.61	0.03	2.23	3.48	5.82	3.43	1.89	1.8	2.1	0	1.87	2.86	1.55	2.29	1.81	0.16	2.3	0.74	2.19
Mutant	L2-341	L2-342	L2-343	L2-344	L2-345	L2-346	L2-347	L2-348	L2-349	L2-350	L2-351	L2-352	L2-353	L2-354	L2-355	L2-356	L2-357	L2-358	L2-359	L2-360
EGFP (+)%	2.43	1.77	3.52	1.73	3.68	3.2	1.88	3.97	4.51	2.37	2.35	0.01	2.87	3.48	2.53	0.99	2.08	0.63	1.16	4.53
Mutant	L2-361	L2-362	L2-363	L2-364	L2-365	L2-366	L2-367	L2-368	L2-369	L2-370	L2-371	L2-372	L2-373	L2-374	L2-375	L2-376	L2-377	L2-378	L2-379	L2-380
EGFP (+)%	3.65	0.14	0.59	1.51	0	2.16	0.72	3.59	3.1	7	1.1	5.35	5.68	3.48	4.44	1.59	4.64	5.09	3.85	3.26
Mutant	L2-381	L2-382	L2-383	L2-384	L2-385	L2-386	L2-387	L2-388	L2-389	L2-390	L2-391	L2-392	L2-393	L2-394	L2-395	L2-396	L2-397	L2-398	L2-399	L2-400
EGFP (+)%	3.8	3	2.2	7.57	0.14	2.95	2.61	3.03	1.87	4.17	0.32	5.29	0.76	1.66	3.51	3.65	0	2.14	7.85	2.13
Mutant	L2-401	L2-402	L2-403	L2-404	L2-405	L2-406	L2-407	L2-408	L2-409	L2-410	L2-411	L2-412	L2-413	L2-414	L2-415	L2-416	L2-417	L2-418	L2-419	L2-420
EGFP (+)%	0.89	2.73	0.05	3.75	3.84	2.18	7.85	3.38	2.7	3.73	2.33	1.67	1.28	3.08	3.3	3.12	0.74	3.96	3.71	2.45
Mutant	L2-421	L2-422	L2-423	L2-424	L2-425	L2-426	L2-427	L2-428	L2-429	L2-430	L2-431	L2-432	L2-433	L2-434	L2-435	L2-436	L2-437	L2-438	L2-439	L2-440
EGFP (+)%	0.58	1.94	0.01	1.39	4.2	0.06	1.86	0.37	2.72	1.35	1.48	1.06	1.57	5.85	2.44	0.08	5.61	2.58	3.42	2.22
Mutant	L2-441	L2-442	L2-443	L2-444	L2-445	L2-446	L2-447	L2-448	L2-449	L2-450	L2-451	L2-452	L2-453	L2-454	L2-455	L2-456	L2-457	L2-458	L2-459	L2-460
EGFP (+)%	2.3	2.13	3.89	1.45	2.73	3.71	1.29	5.34	2.97	0	1.02	2.62	4.84	8.26	7.99	1.05	5.73	0.99	1.11	2.38
Mutant	L2-461	L2-462	L2-463	L2-464	L2-465	L2-466	L2-467	L2-468	L2-469	L2-470	L2-471	L2-472	L2-473	L2-474	L2-475	L2-476	L2-477	L2-478	L2-479	L2-480
EGFP (+)%	0.84	0	1.53	0.53	2.13	2.29	2.37	1.3	1.44	1.67	0.49	4.12	1.44	3.35	6.18	1.97	3.65	4.14	0.83	1.99
Mutant	L2-481	L2-482	L2-483	L2-484	L2-485	L2-486	L2-487	L2-488	L2-489	L2-490	L2-491	L2-492	L2-493	L2-494	L2-495	L2-496	L2-497	L2-498	L2-499	L2-500
EGFP (+)%	0.7	0.01	1.93	2.79	2.83	2.19	0	0	2.43	3.49	2.16	2.04	2.6	0.21	1.02	3.54	4	4.56	2.47	3.7
Mutant	L2-501	L2-502	L2-503	L2-504	L2-505	L2-506	L2-507	L2-508	L2-509	L2-510	L2-511	L2-512	L2-513	L2-514	L2-515	L2-516	L2-517	L2-518	L2-519	L2-520
EGFP (+)%	4.97	2.54	0	0	1.7	1.75	1.72	4.48	2.18	2.54	1.04	2.86	2.59	0	1.88	1.95	3.23	1.54	2.5	2.91
Mutant	L2-521	L2-522	L2-523	L2-524	L2-525	L2-526	L2-527	L2-528	L2-529	L2-530	L2-531	L2-532	L2-533	L2-534	L2-535	L2-536	L2-537	L2-538	L2-539	L2-540
EGFP (+)%	2.74	2.4	3.12	2.47	4.15	1.32	0.05	3.57	0.56	1.46	0	1.33	6.54	5.26	7.8	2.83	5.42	2.53	0.7	4.13
Mutant	L2-541	L2-542	L2-543	L2-544	L2-545	L2-546	L2-547	L2-548	L2-549	L2-550										
EGFP (+)%	3.25	3.23	4.37	10.85	1.54	4.17	4.26	3.94	0.78	1.14										
