# Supplementary material for: Human cell based directed evolution of adenine base editors with improved efficiency
Source: Nat Commun. 2021 Oct 8;12:5897. doi: 10.1038/s41467-021-26211-0 (PMC8501064; doi:10.1038/s41467-021-26211-0)
Supplement: Supplementary file 2 — Reporting Summary [file 41467_2021_26211_MOESM2_ESM.pdf]

nature research

Corresponding author(s): Feng Gu, Jinsong Li

Last updated by author(s): Sep 8, 2021

Reporting Summary

Nature Research wishes to improve the reproducibility of the work that we publish. This form provides structure for consistency and transparency in reporting. For further information on Nature Research policies, see our Editorial Policies and the Editorial Policy Checklist.

Statistics

For all statistical analyses, confirm that the following items are present in the figure legend, table legend, main text, or Methods section.

n/a	Confirmed	
☐	☒	The exact sample size (n) for each experimental group/condition, given as a discrete number and unit of measurement
☐	☒	A statement on whether measurements were taken from distinct samples or whether the same sample was measured repeatedly
☐	☒	The statistical test(s) used AND whether they are one- or two-sided<br>*Only common tests should be described solely by name; describe more complex techniques in the Methods section.*
☒	☐	A description of all covariates tested
☒	☐	A description of any assumptions or corrections, such as tests of normality and adjustment for multiple comparisons
☐	☒	A full description of the statistical parameters including central tendency (e.g. means) or other basic estimates (e.g. regression coefficient) AND variation (e.g. standard deviation) or associated estimates of uncertainty (e.g. confidence intervals)
☐	☒	For null hypothesis testing, the test statistic (e.g. F, t, r) with confidence intervals, effect sizes, degrees of freedom and P value noted<br>*Give P values as exact values whenever suitable.*
☒	☐	For Bayesian analysis, information on the choice of priors and Markov chain Monte Carlo settings
☒	☐	For hierarchical and complex designs, identification of the appropriate level for tests and full reporting of outcomes
☒	☐	Estimates of effect sizes (e.g. Cohen's d, Pearson's r), indicating how they were calculated

Our web collection on statistics for biologists contains articles on many of the points above.

Software and code

Policy information about availability of computer code

Data collection	Illumina MiSeq Control Software (v3.1), BD FACSArial II
Data analysis	Graphpad Prism 8.0.1 (244), Crispresso (version 1.0.10), FACSDiva Version 6.1.3

For manuscripts utilizing custom algorithms or software that are central to the research but not yet described in published literature, software must be made available to editors and reviewers. We strongly encourage code deposition in a community repository (e.g. GitHub). See the Nature Research guidelines for submitting code & software for further information.

Data

Policy information about availability of data

All manuscripts must include a data availability statement. This statement should provide the following information, where applicable:

- Accession codes, unique identifiers, or web links for publicly available datasets
- A list of figures that have associated raw data
- A description of any restrictions on data availability

Next-generation sequencing data underlying all experiments are deposited in the NCBI Sequence Read Archive (PRJNA714183, PRJNA713866).

Field-specific reporting

Life sciences study design

All studies must disclose on these points even when the disclosure is negative.

Sample size	The exact sample size for each experimental group is provided.
Data exclusions	No data were excluded from the study.
Replication	All the confirmation experiments were collected in at least two independent biological replicates.
Randomization	All subjects of study were randomly allocated into experimental groups.
Blinding	The investigators were blinded to the group allocation.

Reporting for specific materials, systems and methods

We require information from authors about some types of materials, experimental systems and methods used in many studies. Here, indicate whether each material, system or method listed is relevant to your study. If you are not sure if a list item applies to your research, read the appropriate section before selecting a response.

Materials & experimental systems

n/a	Involved in the study	
☐	☒	Antibodies
☐	☒	Eukaryotic cell lines
☒	☐	Palaeontology and archaeology
☐	☒	Animals and other organisms
☒	☐	Human research participants
☒	☐	Clinical data
☒	☐	Dual use research of concern

Methods

n/a	Involved in the study	
☒	☐	ChIP-seq
☐	☒	Flow cytometry
☒	☐	MRI-based neuroimaging

Antibodies

Antibodies used	Cas9 Rabbit pAb (ABclonal, A14997), β-Actin Rabbit mAb (ABclonal,AC026)
Validation	Cas9 Rabbit pAb: application (WB, Co-IP), species (Mus musculus , Bos taurus, Homo sapiens), citation"MiCas9 increases large size gene knock-in rates and reduces undesirable on-target and off-target indel edits";<br>β-Actin Rabbit mAb: application (WB, IP,IHC,Co-IP,FC,IF), species (rat brain cells , Pig lung , 293T , DF-1 , Danio rerio , Mus musculus , Homo sapiens , Rattus norvegicus etc.), citation"DNA damage triggers tubular endoplasmic reticulum extension to promote apoptosis by facilitating ER-mitochondria signaling";

Eukaryotic cell lines

Policy information about cell lines

Cell line source(s)	HEK-293 cells: American Type Cell Culture Collection (ATCC)
Authentication	The cell line was authenticated by ATCC.
Mycoplasma contamination	Tested negative for mycoplasma contamination by the supplier, ATCC.
Commonly misidentified lines<br>(See ICLAC register)	The cell line used is not listed as commonly misidentified.

Animals and other organisms

Policy information about studies involving animals; ARRIVE guidelines recommended for reporting animal research

Laboratory animals	B6D2F1(C57BL/6 X DBA2) females: age 7-9 weeks;<br>C57BL/6 males: age 9-14 weeks;<br>ICR females: age 8-10 weeks;
Wild animals	The study did not involve wild animals.
Field-collected samples	The study did not involve field-collected samples.

Ethics oversight	All animal procedures were carried out in accordance with the current guidelines of the Institutional Animal Care and Use Committee (IACUC) at the Center for Excellence in Molecular Cell Science, Shanghai Institute of Biochemistry and Cell Biology, Shanghai, China.

Note that full information on the approval of the study protocol must also be provided in the manuscript.

Flow Cytometry

Plots

Confirm that:

- [x] The axis labels state the marker and fluorochrome used (e.g. CD4-FITC).
- [x] The axis scales are clearly visible. Include numbers along axes only for bottom left plot of group (a 'group' is an analysis of identical markers).
- [x] All plots are contour plots with outliers or pseudocolor plots.
- [x] A numerical value for number of cells or percentage (with statistics) is provided.

Methodology

Sample preparation	Cell culture and transfection procedures are described in the online methods. Cells were washed and filtered through a 30µm cell strainer cap before sorting (36-40h after transfection).
Instrument	BD FACSAriaIII
Software	FACSDiva Version 6.1.3 software was used for analysis.
Cell population abundance	The abundance of cells for flow cytometry analysis was 10,000 for each sample.
Gating strategy	Negative control (untreated) and fluorophore-positive cells were used to establish gates for each cell type. Gates were drawn to collect cells expressing either fluorophore. See the provided examples for gates used.

- [x] Tick this box to confirm that a figure exemplifying the gating strategy is provided in the Supplementary Information.
